# A Novel Aminomethacrylate-Based Copolymer for Solubility Enhancement—From Radical Polymer Synthesis to Manufacture and Characterization of Amorphous Solid Dispersions

**DOI:** 10.3390/polym14071281

**Published:** 2022-03-22

**Authors:** Fabian-Pascal Schmied, Alexander Bernhardt, Christian Moers, Christian Meier, Thomas Endres, Sandra Klein

**Affiliations:** 1 Institute of Biopharmaceutics and Pharmaceutical Technology, Department of Pharmacy, University of Greifswald, Felix-Hausdorff-Straße 3, 17489 Greifswald, Germany; fabian-pascal.schmied@stud.uni-greifswald.de or; 2 Evonik Operations GmbH, Research, Development & Innovation, Kirschenallee, 64293 Darmstadt, Germany; alexander.bernhardt@evonik.com (A.B.); christian.moers@evonik.com (C.M.); thomas.endres@evonik.com (T.E.); 3 Evonik Operations GmbH, Research, Development & Innovation, Rodenbacher Chaussee 4, 63457 Hanau, Germany; christian.meier@evonik.com

**Keywords:** drug release, molecular weight, glass transition, poorly soluble drugs, in vitro dissolution, hot melt extrusion, amorphous solid dispersion, celecoxib, fenofibrate, efavirenz

## Abstract

The present study covers the synthesis, purification and evaluation of a novel aminomethacrylate-based copolymer in terms of its suitability for improving the solubility and in vitro release of poorly water-soluble drug compounds. The new copolymer was synthesized by solvent polymerization with radical initiation and by use of a chain transfer agent. Based on its composition, it can be considered as a modified type of dimethylaminoethyl methacrylate-butyl methacrylate-methyl methacrylate “EUDRAGIT^®^ E PO” (ModE). ModE was specifically developed to provide a copolymer with processing and application properties that exceed those of commercially available (co-)polymers in solubility enhancement technologies where possible. By varying the concentration of the chain transfer agent in the radical polymerization process, the molecular weight of ModE was varied in a range of 173–305 kDa. To evaluate the solubility-enhancing properties of ModE, a series of drug-loaded extrudates were prepared by hot melt extrusion using the novel—as well as several commercially available—(co-)polymers. These extrudates were then subjected to comparative tests for amorphousness, solubility-enhancing properties, storage stability, and drug release. Celecoxib, efavirenz, and fenofibrate were used as model drugs in all experiments. Of all the (co-)polymers included in the study, ModE with a molecular weight of 173 kDa showed the best performance in terms of desired properties and was shown to be particularly suitable for preparing amorphous solid dispersions (ASDs) of the three model drugs, which in a first set of dissolution experiments showed better release behavior under pH conditions of the fasting stomach than higher molecular weight ModE types, as well as a variety of commercially available (co-)polymers. Therefore, the results demonstrate the successful synthesis of a new copolymer, which in future studies will be investigated in more detail for universal application in the field of solubility enhancement.

## 1. Introduction

Addressing the solubility problem of poorly water-soluble drugs is of particular importance for current research into new active pharmaceutical ingredients [1,2,3,4,5]. The number of potential drug candidates exhibiting poor aqueous solubility, that may lead to insufficient drug absorption in the gastrointestinal tract and subsequently to low bioavailability, has increased considerably in recent years [3,5,6,7,8]. To overcome the challenge of a drug’s low aqueous solubility, strategies such as particle size reduction, complexation using cyclodextrins, self-emulsifying systems or amorphization leading to supersaturation of the drug in aqueous media can be used to increase the solubility and/or dissolution rate of poorly water-soluble drugs [3,8]. The amorphous state is characterized by an increased free energy, which can lead to an improved solubility of the drug and thus also improve its bioavailability after oral administration compared to the corresponding crystalline active substance [3,5,8,9]. Recrystallization of the drug from a metastable, amorphous state is widely reported to have an adverse effect on its solubility in aqueous fluids [3,7,8,9]. To maintain the amorphous state, as well as to prevent recrystallization, drugs are usually embedded in a polymer matrix [9,10].

Several (co-)polymers—such as polyvinyl caprolactam-polyvinyl acetate-polyethylene glycol graft copolymer, polyvinylpyrrolidone-polyvinyl acetate copolymer, dimethylaminoethyl methacrylate-butyl methacrylate-methyl methacrylate, different types of hydroxypropyl methylcellulose (HPMC), as well as hydroxypropyl methylcellulose acetate succinate (HPMCAS)—have proven suitable for the purpose of enhancing the solubility of poorly water-soluble drugs [3,7,9,10,11]. EUDRAGIT^®^ E PO (EPO) is a copolymer that consists of the three monomers dimethylaminoethyl methacrylate, butyl methacrylate and methyl methacrylate in a ratio of 2:1:1 by weight. It has been a commercial product for several decades and is widely used in many applications, including solubility enhancement strategies [12]. EPO is insoluble in saliva, but soluble in fasted gastric fluid (pH < 5) enabling immediate drug release in the stomach to ensure fast drug absorption in the upper intestine. However, if one wants to use EPO for the preparation of drug-loaded ASDs, this often proves challenging, as it is a flexible copolymer with a moderately high glass transition (T_g_), which allows strong movement of the polymer chains. Consequently, when applying high drug loads, a lower storage stability of the formulations due to a further decrease of the copolymers T_g_ and subsequent recrystallization of the drug can become a major concern [13]. However, some limitations and drawbacks of EPO related to this issue might be compensated by structural modifications. One approach to create a functionally similar but more suitable copolymer for the preparation of stable ASDs is to substitute one or more of the monomers used for EPO synthesis with structurally-related monomers that would contribute to a higher T_g_ in the final copolymer [14]. Likewise, altering the molecular weight (M_w_) of the copolymer might be beneficial [14,15]. Both a change in monomer composition and the aim of changing the molecular weight of the polymer to be produced usually require a change in polymer synthesis and reaction conditions [14,16].

The M_w_ of a (co-)polymer can be adjusted by different means, for instance, by altering the drip rate of the radical initiator added to the monomer mixture or by adding different concentrations of a chain-transfer-agent (CTA). A CTA is a molecule that has at least one weak chemical bond and that, when added to the reaction mixture, interrupts chain growth by reacting with the free-radical site of a growing polymer chain. CTAs are usually added to control the chain length during the synthesis of a polymer to achieve the desired mechanical and processing properties [15,17]. The impact of CTAs on the average M_w_ of a (co-)polymer to be synthesized is concentration dependent and the M_w_ of the resulting (co-)polymer typically decreases with increasing CTA concentration. Regardless of whether a CTA is added or not, it is desirable to achieve a high monomer conversion by choosing suitable reaction conditions for the polymerization process, which at the same time promises a very low number of residual monomers [18]. Altogether, this would not only increase the yield, but also reduce the time required for the purification of the (co-)polymer. By means of a robust synthesis method, (co-)polymers which reflect the mass ratio of the monomers used, and consequently also the ratio of their functional units, can be obtained in a reproducible way [18].

The aim of the present study was the reproducible synthesis of an aminomethacrylate-based copolymer with the lowest possible number of residual monomers. Structural modification of EPO should result in a modified EPO copolymer (ModE) consisting of about 50 wt% of its amino-functional unit, which is suitable for improving the dissolution rate and the solubility of poorly water-soluble drugs by preparing drug-loaded ASDs. The ASDs produced using the new copolymer and different model drugs should be characterized by a pronounced storage stability; therefore, the effect of varying T_g_ and M_w_ of the copolymer on this property should be investigated.

## 2. Materials and Methods

### 2.1. Materials

Celecoxib obtained from Aarti Drugs Ltd. (Mumbai, India), efavirenz received from Angene International Ltd. (London, UK) and fenofibrate provided by D.K. Pharma Chem PVT Ltd. (Maharashtra, India) were used as model compounds. Dimethylaminopropyl methacrylamide was purchased from TCI Deutschland GmbH (Eschborn, Germany). Butyl methacrylate and methyl methacrylate were provided by Röhm GmbH (Darmstadt, Germany). Tert-butyl peroxyneodecanoate was received from Nouryon (Amsterdam, The Netherlands) and tert-butyl peroxypivalate was obtained from United Initiators GmbH & Co. KG (Pullach im Isartal, Germany). Polyvinyl caprolactam-polyvinyl acetate-polyethylene glycol graft copolymer (Soluplus^®^, M_w_ = 90,000–140,000 g/mol), polyvinylpyrrolidone-polyvinyl acetate copolymer (Kollidon^®^ VA 64, M_w_ = 45,000–70,000 g/mol) and polyvinylpyrrolidone (Kollidon^®^ 17 PF, M_w_ = 7000–11,000 g/mol) were purchased from BASF SE (Ludwigshafen, Germany). Hydroxypropyl methylcellulose acetate succinate (AQOAT^®^ AS-MMP, M_w_ = 18,000 g/mol) was kindly donated by Shin-Etsu Chemical Co., Ltd. (Tokio, Japan). Hydroxypropyl methylcellulose (Affinisol^®^ HPMC 100 LV, M_w_ = 179,000 g/mol) was provided by Dow Chemical Company (Schwalbach am Taunus, Germany). Dimethylaminoethyl methacrylate-butyl methacrylate-methyl methacrylate (EUDRAGIT^®^ E PO, EUDRAGIT^®^ E 100, M_w_ = 47,000 g/mol) were from Evonik Operations GmbH (Darmstadt, Germany). n-propanol and n-dodecylmercaptan were obtained from Merck KGaA (Darmstadt, Germany). All other chemicals were of analytical grade and purchased commercially.

### 2.2. Methods

#### 2.2.1. Preparation of ModE Copolymer

For the preparation of ModE copolymer (dimethylaminopropyl methacrylamide-butyl methacrylate-methyl methacrylate copolymer 2:1:1 (ratios by weight)), the monomers dimethylaminopropyl methacrylamide (500.0 g), butyl methacrylate (250.0 g) and methyl methacrylate (250.0 g) were added into a 3-L round-bottom flask reaction vessel equipped with a propeller stirrer, a reflux condenser and a nitrogen inlet. The reaction vessel was placed in a preheated water bath at 82 °C and the mixture of monomers was stirred at 300 rpm. Immediately after reaching an internal temperature of 65 °C, the CTA n-dodecylmercaptan (either 3.0 g, 5.0 g, 9.0 g or 15.0 g), was added in a 1:1 (*w*:*w*) mixture with n-propanol as a bolus to control the M_w_ of the copolymer during the polymerization process. The different amounts of n-dodecylmercaptan indicated were chosen in order to selectively modify the M_w_ of the synthesized copolymer and then evaluate the effects of the M_w_ on the desired polymer properties and applications. While stirring the mixture in the reaction vessel, the internal temperature was continuously increased until a temperature of 80 °C was reached. Then, 1000 g of a tert-butyl peroxyneodecanoate (0.6 wt%) solution in n-propanol was added with a continuous flow rate of 5 g/min to initiate a radical polymerization as well as to counteract the increasing viscosity throughout the polymerization process. After approximately 3.5 h at a temperature of 80–85 °C, the second initiator, tert-butyl peroxypivalate (0.5 g) was added to complete the reaction within the following 90 min. Thereafter, the mixture was cooled down to 60 °C and transferred to a drying oven with exhaust air at 40 °C to remove n-propanol over 48 h. This drying process provided a coarse copolymer block to which then 1 L of liquid nitrogen was added to enable grinding (mesh size: 0.25 mm) using an Ultra Centrifugal Mill ZM 200 from Retsch GmbH (Haan, Germany). Subsequently, the ground copolymer was subjected to a purification process for the purpose of extracting residual monomers and organic solvent.

#### 2.2.2. Purification and Drying of ModE

For removing residual monomers and the organic solvent n-propanol, the ground ModE was subjected to a purification procedure. For this purpose, it was transferred to a 5-L beaker and 3 L of deionized water were added. Using an overhead stirrer with a perforated stirring blade, the copolymer-water mixture was stirred at 300 rpm for a total of 36 h. During this procedure, the deionized water was completely replaced every 4 h by separating water and copolymer using a sieve (mesh size: 0.1 mm) before fresh water was added again to the copolymer. The purified copolymer was finally dried for 10 days at 50 °C in a drying cabinet. Since after drying, several agglomerates of the copolymer had formed, it was ground (mesh size: 0.25 mm) one more time using the Ultra Centrifugal Mill ZM 200.

#### 2.2.3. Determination of Water Content of ModE

The water content of ModE was measured after 5 and 10 days of drying using the Moisture Analyzer HC 103 from Mettler Toledo (Giessen, Germany). This device works according to the thermo-gravimetric principle, often referred to as the loss-on-drying (LOD) principle. The device consisted of two components, a heating as well as a balance unit. In order to determine the LOD, a sample of a defined weight (1 g) was placed on the balance. Then, a halogen lamp, which heated and dried the sample, was switched on. During drying, the sample weight was continually recorded via the integrated balance. A temperature of 105 °C was maintained during the entire measurement. The water content was measured automatically, and the result was displayed by the instrument when no change in sample weight was monitored within 10 consecutive seconds. All measurements were performed in triplicate.

#### 2.2.4. Residual Monomer Analysis and Monomer Conversion Rate

Prior to the removal of volatiles immediately after the polymerization process, and also after the completion of the purification and drying process, samples (n = 3) of ModE were withdrawn for the analysis of residual monomers. High performance liquid chromatography (HPLC) was used for the analysis of dimethylaminopropyl methacrylamide and gas chromatography (GC) for the analysis of butyl methacrylate and methyl methacrylate. Results from residual monomer quantification were then used to calculate the amount of polymerized monomers providing the final copolymer composition. For HPLC analysis, an Agilent 1260 Infinity system from Agilent Technologies (Frankfurt am Main, Germany) was used. The GC analysis was conducted using a Clarus 500 GC with a head space sampler (Turbomatrix 40), both from PerkinElmer LAS GmbH (Rodgau, Germany).

#### 2.2.5. Molecular Weight Analysis by Gel Permeation Chromatography

The molecular weight distribution of ModE was determined by gel permeation chromatography (GPC) using an Agilent 1100 Series GPC-SEC Analysis System comprising a pump (G1310A), autosampler (G1313A), column oven (G1316A), RI-detector (G1362A) and a control module (G1323B), all from Agilent Technologies (Frankfurt am Main, Germany). Separation was achieved using a GRAM precolumn (8 × 50 mm, 10 µm) and another three GRAM columns (8 × 300 mm, 10 µm) in series, all maintained at 60 °C. The eluent consisted of n,n-dimethylacetamide: lithium bromide: tris(hydroxymethyl)aminomethane (TRIS): water (1000:2:2:10 *w*/*w*), the flow rate was set to 1 mL/min and an injection volume of 100 µL was applied. The RI-detector was maintained at 40 °C and a polymethylmethacrylate solution (1 g/L eluent) was used as a standard. A solution of ModE (1 g/L eluent) was used for analysis and the number-average molecular weight (M_n_), the weight-average molecular weight (M_w_), and the polydispersity index (PDI) of ModE was determined in triplicate.

#### 2.2.6. Thermogravimetric Analysis for Studying ModE Decomposition

In order to obtain information on whether the new polymer can be processed with the application of heat without decomposition, which is for instance required for melt extrusion, an isothermal thermogravimetric analysis (TGA) of ModE was performed to measure weight changes of ModE as a function of temperature and time using a TGA Q5000 from TA Instruments (Huellhorst, Germany). Analyses were performed as follows: samples of 20 mg each were placed into the device’s sample chamber and preheated for 15 min at a temperature of 100 °C to remove residual water. Thereafter, samples were kept at a fixed temperature of either 150 °C or 165 °C for another 25 min and the copolymers’ change in weight was continuously recorded.

#### 2.2.7. Flowability Measurement

The flowability of the ground copolymers ModE and EPO was analyzed using the flowability tester BEP 2 from Copley Scientific (Nottingham, UK). Both the time required for 100 g of each of the copolymers to flow through a 10 mm nozzle and the slope angle of the resulting pile was measured. A high flow rate as well as a low slope angle (<40°) indicated a good flowability. For better comparability, prior to the analysis EPO was also reduced to a particle size of approximately 0.25 mm using the Ultra Centrifugal Mill ZM 200. The flowability measurements were conducted in triplicate.

#### 2.2.8. Preparation of Amorphous Solid Dispersions via Hot Melt Extrusion

To evaluate the suitability of the new ModE copolymer for the preparation of solid ASDs, various ModE copolymers with different M_w_ were extruded with different model drugs, i.e., celecoxib, efavirenz or fenofibrate, by hot melt extrusion (HME). For comparison, using the same manufacturing process, ASDs were prepared from the same model drug substances with (co-)polymers already established for the purpose of solubility enhancement such as EPO, Soluplus^®^, Kollidon^®^ VA 64, Kollidon^®^ 17 PF, AQOAT^®^ AS-MMP and Affinisol^®^ HPMC 100 LV. Extrudates were prepared as follows: first, 15 g of a mixture of each of the individual (co-)polymers and one of the poorly soluble model drugs celecoxib, efavirenz, or fenofibrate was prepared in a predetermined mixing ratio by mixing the components in a 100 mL jar sealed with a screw cap for 10 min using a turbular mixer (TURBULA^®^, WAB Group, Nidderau-Heldenbergen, Germany). Then, the polymer-drug blends were processed via HME technology to obtain ASDs using a co-rotating HAAKE MiniLab twin screw extruder with a conical screw design from Thermo Fisher Scientific (Dreieich, Germany). The HME process was characterized by the set parameters of screw speed and process temperature, and by the torque recorded during the process. The die diameter was 2 mm, and the strand leaving the extruder was allowed to cool during transport via a conveyor belt and was finally ground to coarse granules by a small chopper with a rotating metal gear. The coarse granules were then pulverized using the ZM 200 ultra-centrifugal mill (mesh size: 0.25 mm). The obtained powders were used for all further experiments.

#### 2.2.9. Differential Scanning Calorimetry Analysis

All ASDs were analyzed via differential scanning calorimetry (DSC) to determine whether the incorporated drug was in the amorphous or crystalline state. All DSC analyses were conducted using a DSC 3+ (DSC-HC01) from Mettler Toledo (Giessen, Germany). A sample of 5–10 mg each was weighed into a small, aluminum pan with a perforated lid and exposed to a heating-cooling-heating cycle in a temperature range of 0 °C to 200 °C. The heating/cooling rate was set at 10 °C/min and a nitrogen flow of 50 mL/min was applied while running the measurement. For comparison, the melting point of the pure drug substance as well as the glass transition temperature of the individual (co-)polymers were investigated. For all analyzed samples, T_g_ was taken from the thermogram obtained from the second heating cycle, and the indicated value represents the mean of n = 3.

#### 2.2.10. X-ray Powder Diffraction Studies

X-ray powder diffraction (XRPD) studies were performed to verify the results of the DSC analysis. For these investigations, all individual samples, i.e., drug substances, (co-)polymers and all ASDs, were first triturated to a fine soft powder in a ceramic mortar, and 500 mg of each sample was packed (back-loaded) into the 16 mm-diameter well of a sample holder. Analyses were then run on a Cubix3 Pharma diffractometer from Malvern Panalytical (Malvern, UK) in Bragg–Brentano geometry using the following components and parameters: X-ray tube: LFF-Cu X-ray tube, Cu Kα, λ = 0.1542 nm, detector: X’Celerator, generator settings: 40 mA, 40 kV, rotation: 1 revolution/s, 2-Theta(θ) range: 2°–40° with 0.02° steps, applying 200 s per step. The intensity (count) of the diffracted X-rays was recorded as a function of the diffraction angle θ and the resulting diffractogram was displayed as intensity (count) against 2θ. The positions of the angles were determined by the geometry of the unit cell of the crystalline phase. The relative intensities (counts) of the peaks observed were modulated by different diffraction behavior of atoms with different electrons’ density function (atomic number) and by the positions of the individual atoms in the unit cell.

#### 2.2.11. Fourier-Transform Infrared Spectroscopy Analysis

Fourier-transform infrared spectroscopy (FT-IR) analysis was performed to investigate potential molecular interactions between the individual model drugs and the ModE copolymer. Therefore, drug substances, ModE copolymer, a physical mixture (PM) of the drug substance and ModE copolymer, as well as the corresponding ASDs, were analyzed using the FT-IR spectrometer “ALPHA” from Bruker Optics (Hanau, Germany). The samples were placed into the light path for recording the spectra, and the selected scanning range was 4000 to 400 cm^−1^. Characteristic patterns (spikes) related to the chemical structure of the samples were identified by measuring the attenuated total reflection (ATR) of the exposed infrared radiation. The resulting FT-IR spectrogram was obtained by plotting the transmission (%) against the wave number (cm^−1^).

#### 2.2.12. Determination of Saturation Solubility in Water

Saturation solubility of celecoxib, efavirenz and fenofibrate in water was studied for pure drugs and the corresponding ASDs immediately after preparation. For this purpose, approximately 25 mg of the substance or a quantity of ASD corresponding to this amount of drug substance was added to a volume of 25 mL of distilled water and stirred at controlled temperature in a water bath at 20 °C for 48 h (100 rpm). The resulting suspensions were filtered via a 0.22 µm polytetrafluoroethylene (PTFE) membrane filter with a diameter of 25 mm from Global Biomed Scientific (Forest, VA, USA). The appropriateness of the filter material had been validated for each of the drug compounds before use. All filtrates were diluted with acetonitrile in a predetermined ratio, before the amount of celecoxib, efavirenz, and fenofibrate dissolved was quantified by HPLC.

#### 2.2.13. Dissolution Studies of ASD

Dissolution experiments were conducted in triplicate with 25 mg drug substance or an equivalent amount of ASD using USP apparatus II (DT 800 LH) from ERWEKA GmbH (Langen, Germany). The paddle speed was set to 100 rpm to avoid coning effects and all experiments were performed in 500 mL of 0.1 M hydrochloric acid at 37 ± 0.5 °C. The test duration was 120 min. All samples were withdrawn by a fraction collector, equipped with poroplast cannula filters of 10 µm pore size and manually diluted 1:1 (*v*/*v*) with acetonitrile, before HPLC analysis.

#### 2.2.14. HPLC Setup

An HPLC system (Agilent 1260 Infinity) was used for the quantification of the three different model drug substances. The system consisted of a quaternary pump (G1311B), an autosampler (G1329B), a column oven (G1316A) and an UV detector (G1314C), all from Agilent Technologies (Frankfurt am Main, Germany). Prior to use, all analytical methods were validated according to USP requirements.

##### HPLC Method for Celecoxib

Separation of all samples containing celecoxib was achieved using a Knauer Nucleosil 100-7 C18 (125 × 4.6 mm, 7 µm) column (Knauer-Wissenschaftliche Geräte GmbH, Berlin, Germany) maintained at 40 °C. The mobile phase consisted of an acetonitrile: water: triethylamine mixture (300:300:0.9 *v*/*v*), adjusted to pH 3.00 with phosphoric acid. The flow rate was set to 1.8 mL/min. An injection volume of 5 µL was applied, the run time was 7 min, and celecoxib was detected at 254 nm. In the concentration range of 0.13–542 µg/mL, the analytical curve was linear (r^2^ = 0.999995). The method was found to be accurate (100.2–102.1%) and precise (CV 2.46%) with a quantification limit of 0.05 µg/mL.

##### HPLC Method for Efavirenz

Separation of the efavirenz samples was achieved using a Symmetry 300 C18 (250 × 4.6 mm, 5 µm) column (Waters GmbH, Eschborn, Germany) maintained at 22 °C. The mobile phase consisted of an acetonitrile: buffer solution (disodium hydrogen phosphate/phosphoric acid adjusted to pH 3.60) mixture (290:210 *v*/*v*). The flow rate was set to 1.5 mL/min and the injection volume was 20 µL. The run time was 10 min and efavirenz was detected at 247 nm. In the concentration range of 0.13–515 µg/mL, the analytical curve was linear (r^2^ = 0.999894). The method was found to be accurate (101.4–103.0%) and precise (CV 4.05%), with a quantification limit of 0.05 µg/mL.

##### HPLC Method for Fenofibrate

Separation of the fenofibrate samples was achieved on a Symmetry 300 C18 (150 × 4.6 mm, 5 µm) column maintained at 22 °C. The mobile phase consisted of an acetonitrile: water mixture (70:30 *v*/*v*), adjusted to pH 2.50 with phosphoric acid. The flow rate was set to 2.0 mL/min. The injection volume was set at 20 µL, the run time was 6 min and fenofibrate was detected at 286 nm. In the concentration range of 0.13–526 µg/mL, the analytical curve was linear (r^2^ = 0.999992). The method was found to be accurate (101.2–101.4%) and precise (CV 2.42%) with a quantification limit of 0.05 µg/mL.

For all three methods, selectivity was determined (formulation excipients), and no interference was observed in the retention time of celecoxib, efavirenz, or fenofibrate. In addition, the peak area of the specific drug did not change in the presence of all excipients used in the study.

#### 2.2.15. Stability Studies

ASDs were added to 30 mL amber-glass jars, closed with a screw cap, and stored at constant and controlled conditions (30 °C/65% RH) in a climatic chamber from Binder GmbH (Tuttlingen, Germany) for three months. After three months, they were first visually inspected to determine whether they could be easily fluffed up again or stuck together (formation of larger agglomerates). Subsequently, they were subjected to dissolution experiments and DSC analyses using the same conditions as selected for the studies immediately after manufacture, and results of these experiments were compared with those obtained immediately after manufacture.

#### 2.2.16. Data Analysis

All reported data were derived from at least three independent experiments. Significance tests were conducted with SigmaPlot 14.0 from Systat Software GmbH (Erkrath, Germany) using a one-way analysis of variance (ANOVA) test with the Holm–Sidak post-test. Significances are indicated with *p* < 0.05 in brackets.

## 3. Results and Discussion

### 3.1. Synthesis of ModE

**Figure 1:**
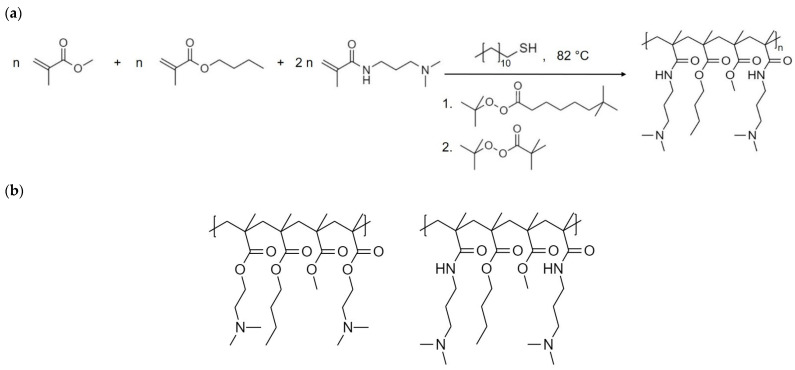
Radical copolymer synthesis of ModE including monomers, chain-transfer-agent (CTA), initiators (1. tert-butyl peroxyneodecanoate, and 2. tert-butyl peroxypivalate) and reaction conditions (**a**), and a section of the chemical structure of EUDRAGIT^®^ E PO (EPO) (left) and ModE (right) (**b**).

ModE, a dimethylaminopropyl methacrylamide-butyl methacrylate-methyl methacrylate copolymer, was synthesized by applying four different concentrations of the CTA n-dodecylmercaptan (0.3%, 0.5%, 0.9% and 1.5% by weight). The higher the CTA concentration, the lower the M_w_ of the ModE type produced. Four copolymers with different weight average M_w_ of 173 kDa, 254 kDa, 281 kDa and 305 kDa were obtained, which will be referred to in the following as copolymers E-173 kDa, E-254 kDa, E-281 kDa and E-305 kDa. Figure 1a illustrates the radical copolymer synthesis of ModE, and Figure 1b shows the differences in the chemical structure of ModE and EPO. Overall, ModE was obtained by replacing the functional monomer unit of EPO—more precisely the dimethylaminoethyl methacrylate by dimethylaminopropyl methacrylamide.

### 3.2. Purification, Residual Monomer Analysis and Water Content of ModE

Based on the results of the residual monomer analysis immediately after polymer synthesis (on average 6.16 wt% for dimethylaminopropyl methacrylamide, 0.002 wt% for butyl methacrylate, and 0.035 wt% for methyl methacrylate), considering a monomer ratio of 2:1:1 (dimethylaminopropyl methacrylamide: butyl methacrylate: methyl methacrylate) by weight, the average monomer conversion rate was calculated as 87.68% for dimethylaminopropyl methacrylamide, 99.99% for butyl methacrylate, and 99.86% for methyl methacrylate (Table 1). Consequently, with the applied radical polymerization process, a high monomer conversion providing a yield >90% was achieved. The results led to a final copolymer composition comprising in average 46.74 wt% dimethylaminopropyl methacrylamide, 26.65 wt% butyl methacrylate, and 26.61 wt% methyl methacrylate (Table 1). Regardless of its M_w_, ModE presented with a proportion of its amino methacrylamide functional unit of about 47 wt% which is very close to the targeted value of 50 wt%. The proportion of amino functionality is therefore comparable to that of EPO.

The residual monomer content of butyl methacrylate and methyl methacrylate had already been very low immediately after radical polymerization synthesis but could be further reduced by applying a water-based purification process. In particular, the residual monomer content of the dimethylaminopropyl methacrylamide monomer could be significantly (*p* < 0.05) reduced by this purification step. Independent of their M_w_, all ModE copolymers finally exhibited a total residual monomer content below 0.05% (Table 2).

The residual water content of all ModE copolymers (Table 2) was monitored after 5 and 10 days of drying, respectively. Results indicate that the target residual water content of less than 1% was achieved after 10 days of drying. It was assumed that this specified maximum acceptable water content would effectively prevent agglomeration that could affect processability.

### 3.3. Molecular Weight Analysis

Results from the GPC analysis of the four ModE copolymers obtained by utilizing different concentrations of the CTA n-dodecylmercaptan in copolymer synthesis (Table 3) indicated that a higher concentration of the CTA led to a decrease of both the M_n_ and the M_w_, as expected for this radical polymerization. By contrast, the PDI slightly increased with increasing CTA concentration (Table 3).

### 3.4. Flowability

Since a good flowability of a powder is an essential prerequisite for easy processing and dose accuracy, immediately after grinding, the flowability of ModE copolymers was measured. With increasing M_w_, and inversely with decreasing PDI, the flowability of ModE improved (Table 4). As all copolymers had been ground to a similar particle size of about 0.25 mm immediately before running the flowability test, an impact of the particle size could be neglected. Independent of their M_w_, all ModE types demonstrated a slope angle that was substantially smaller than 40°, indicating good flow properties. In contrast to all ModE copolymers, EPO revealed no product flow through the nozzle of the flowability tester.

### 3.5. Investigation on Decomposition of ModE via TGA

The four different types of ModE copolymers were first dried at 100 °C and then subjected to isothermal TGAs at 150 °C and 165 °C, i.e., the HME process temperatures, to mimic the heat input that the copolymers would be subjected to during the investigation. Based on the results of stability studies with EPO, whose chemical structure is very similar to that of ModE, it might be possible that, at the selected temperatures, decomposition of ModE copolymers might take place in the side chain region. Possible decomposition products in this case would be dimethylamine, trimethylamine, acetaldehyde, and short-chain alcohols, i.e., substances that are volatile at these high temperatures. Accordingly, possible decomposition of ModE could be easily detected in the context of isothermal TGA, since this would be associated with a measurable change in weight due to the escape of volatile compounds.

The time ModE would be exposed to temperatures of 150 °C and 165 °C in the extruder would range from 5–10 min. From the TGA thermograms (Figure S1a,b), it is evident that at both temperatures ModE did not decompose noticeably, for a time period of at least 10 min, since the thermograms showed no significant (*p* > 0.05) weight change (<1% after 10 min in each case) for all ModE copolymers tested. As expected, the weight loss at a temperature of 165 °C (Figure S1b) was slightly higher than at 150 °C (Figure S1a). However, even after 25 min of heat exposure, there were no noticeable changes in weight, which is why the copolymer was regarded as suitable for the HME process.

### 3.6. ASD Composition and Manufacture

In the next step, the novel ModE copolymers were used to produce ASDs by HME. For comparative purposes, ASDs were also prepared using other (co-)polymers, including EPO, which are commonly used for solubility enhancement. The total drug load, as well as the process parameters applied in the HME process, are displayed in Table 5. The extrusion temperature was chosen individually for each (co-)polymer based on its melt viscosity characteristics when applying thermal and mechanical force input. A high torque indicates a pronounced melt viscosity, which can lead to clogging of the extruder die if the extruded material cools immediately upon leaving the extruder. Torque values higher than 250 N·cm proved to complicate the HME process. The issue of very high torque values could be addressed by increasing the processing temperature if the corresponding (co-)polymers could tolerate higher temperatures. While the same screw speed was used for all other (co-)polymers, Affinisol^®^ HPMC 100 LV was extruded at a lower screw speed, as it would otherwise have clogged the extruder die. A further increase in extrusion temperature was not an alternative for processing Affinisol^®^ HPMC 100 LV, as this would have led to a loss of integrity. All commercially available (co-)polymers were processed via HME within their recommended temperature range provided by the corresponding manufacturer, if possible [19,20,21]. For the use of these (co-)polymers, there is also information from other HME studies, in which generally somewhat lower extrusion temperatures were used than indicated in Table 5 [22,23,24,25,26]. This is, however, probably due to differences in drug loads and extruder types used, i.e., parameters that have an influence on both the torque values and the temperature required for a successful extrusion process [23,24,25,26].

### 3.7. Thermal Characterization of the Pure Drugs, (Co-)Polymers and ASDs via DSC Analysis

Immediately after processing, celecoxib- (Figure S2a,b), efavirenz- (Figure S3a,b) and fenofibrate ASDs (Figure S4a,b), as well as the individual (co-)polymers (Table 6), were analyzed via DSC (thermograms displayed in endo up mode). ASDs and (co-)polymers showed, without exception, amorphous properties (no melting peaks) (Figures S2–S4), while the crystalline character of the pure drug substances was revealed by characteristic peaks in the respective melting range of the drug substances (Figures S2–S4). Moreover, the T_g_ of the corresponding ASD was always lower than that of the pure (co-)polymer. EPO showed a significantly (*p* < 0.05) lower T_g_ than all other (co-)polymers applied in the study. The T_g_ of EPO was also lower than that of all ASDs prepared with ModE copolymers. Consequently, the T_g_s of the drug loaded EPO ASDs were the lowest of all T_g_s measured, which may lead to storage stability issues related to crystallization of the active ingredient and, accordingly, lower drug release.

### 3.8. XRPD Studies of Pure Drugs, (Co-)Polymers and ASDs

**Figure 2:**
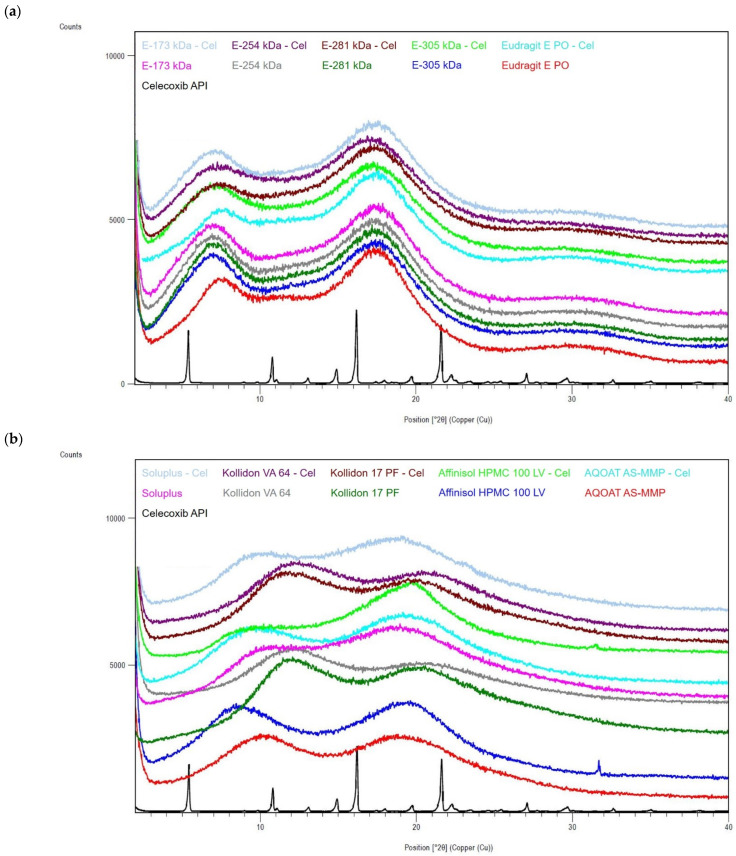
X-ray powder diffraction (XRPD) diffractograms of the drug substance celecoxib, ModE copolymers, EPO and the corresponding ASDs (**a**), as well as celecoxib drug substance, other marketed (co-)polymers (selection) and the corresponding ASDs (**b**).

**Figure 3:**
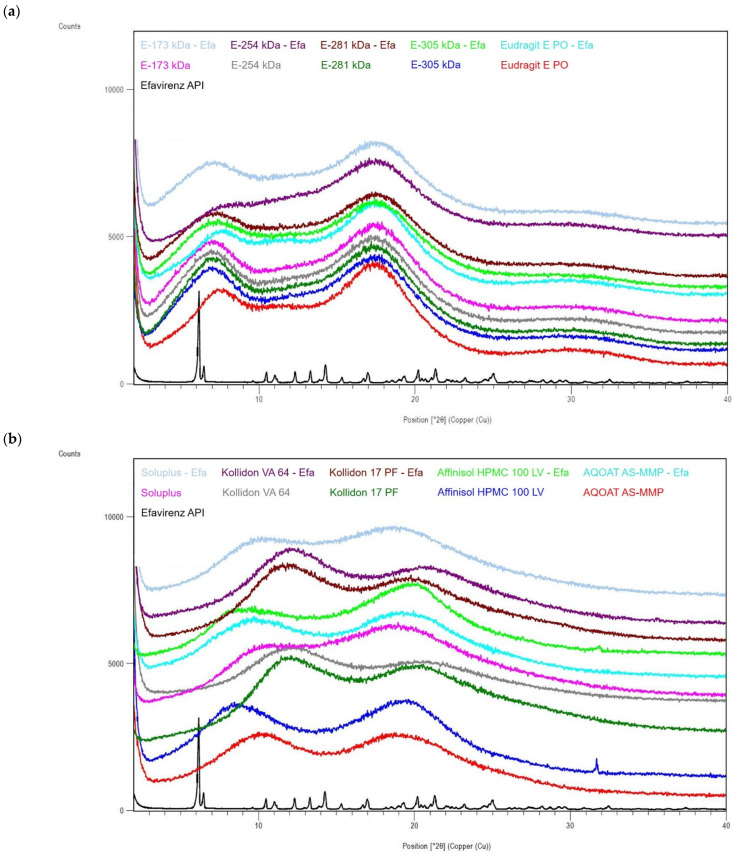
X-ray powder diffraction (XRPD) diffractograms of the drug substance efavirenz, ModE copolymers, EPO and the corresponding ASDs (**a**), as well as efavirenz drug substance, other marketed (co-)polymers (selection) and the corresponding ASDs (**b**).

**Figure 4:**
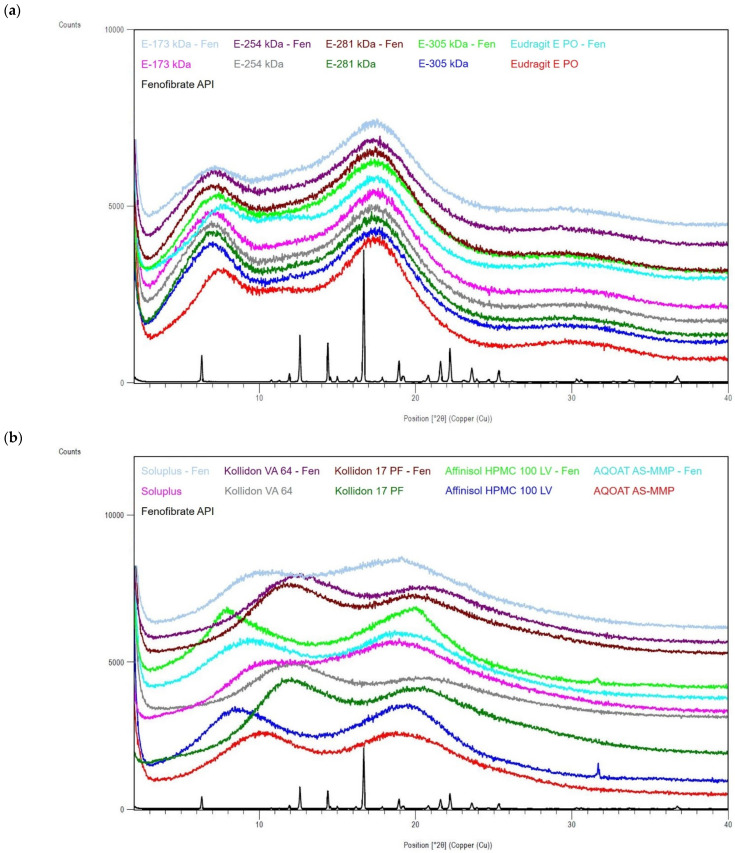
X-ray powder diffraction (XRPD) diffractograms of the drug substance fenofibrate, ModE copolymers, EPO and the corresponding ASDs (**a**), as well as fenofibrate drug substance, other marketed (co-)polymers (selection) and the corresponding ASDs (**b**).

XRPD analyses of the pure drug substances celecoxib (Figure 2a,b), efavirenz (Figure 3a,b) and fenofibrate (Figure 4a,b) provided characteristic diffraction peaks indicating crystalline phases. In contrast, the XRPD patterns of both the pure (co-)polymers and all ASD formulations prepared by HME did not exhibit diffraction peaks, regardless of the incorporated drug substance, indicating that these materials were amorphous (Figure 2, Figure 3 and Figure 4) and proving that the preparation of ASDs by HME was successful in all cases.

### 3.9. FT-IR Analysis

As a representative of all ModE copolymers, E-173 kDa was selected to investigate the potential interactions between the copolymer and the drugs celecoxib, efavirenz, or fenofibrate, which may occur during HME processing. The selection of only one representative for the FT-IR analysis was considered sufficient for this study, since differences between the ModE polymers are mainly limited to their M_w_s.

**Figure 5:**
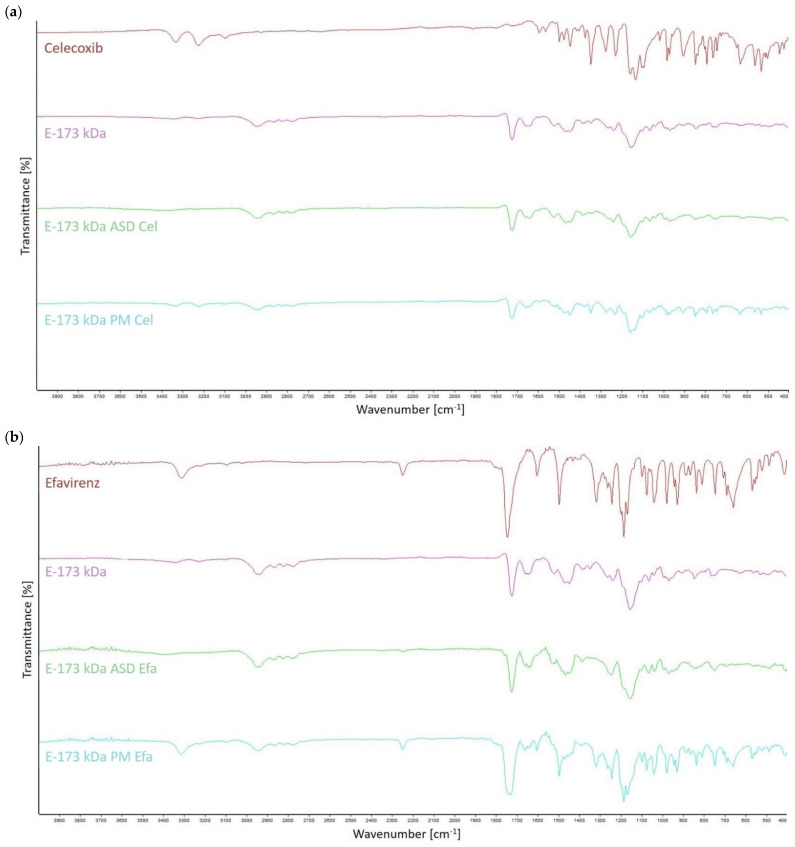

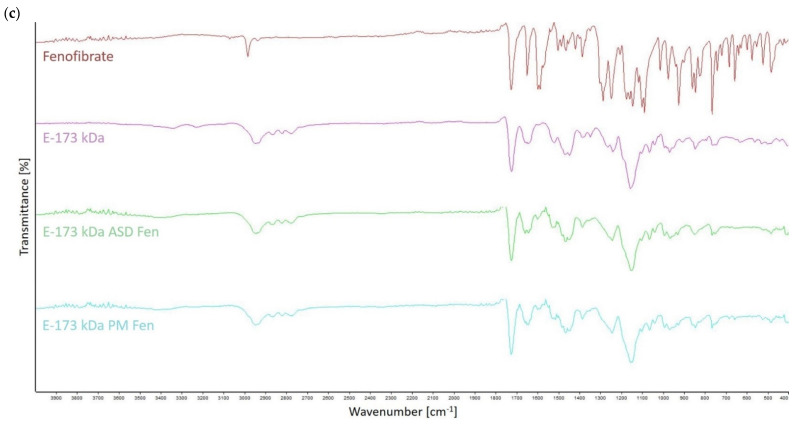
Fourier-transform infrared (FT-IR) spectrogram overlays of celecoxib (**a**), efavirenz (**b**), and fenofibrate (**c**), plotted together with E-173 kDa, a physical mixture (PM) of the specific drug and E-173 kDa, as well as the corresponding ASD processed via hot melt extrusion (HME).

The spectrum of the celecoxib molecule presented with characteristic peaks of the NH_2_ group at 3332 cm^−1^ and 3224 cm^−1^ (N-H stretching vibrations) as a potential H-donor group, as well as a potential H^+^-acceptor in the SO_2_ group at 1345 cm^−1^ (S=O stretching vibrations) (Figure 5a). The copolymer E-173 kDa revealed distinctive peaks between 2950 and 2750 cm^−1^ of its dimethylamino group (N-CH_3_ stretching vibrations), as well as a peak of its carbonyl groups at 1723 cm^−1^ (C=O stretching vibrations) as a potential H^+^-acceptor (Figure 5a). The intensity of the characteristic celecoxib peaks in both the ASD and PM formulation spectra decreased significantly compared to those observed in the spectrum of the pure drug which was due to the lower drug concentration in these samples. The fact that celecoxib peaks between 3400 and 3200 cm^−1^ were no longer detectable in the ASD spectrum, but less pronounced, yet still present in the spectrum of the PM, might be attributable to a potential interaction (via hydrogen bonds) between the proton accepting groups (C=O) in ModE and the proton donating groups (NH) of celecoxib. In addition, the disappearance of the characteristic celecoxib peak at 1345 cm^−1^, which was observed for the ASD but not for the PM formulation (Figure 5a), was considered to indicate a possible further interaction.

The efavirenz spectrum revealed characteristic peaks at 3313 cm^−1^ (N-H stretching vibrations), 2249 cm^−1^ (C≡C stretching vibrations), 1744 cm^−1^ (C=O stretching vibrations) and around 1241 cm^−1^ (C-F stretching vibrations) (Figure 5b). As had been observed for the celecoxib formulations, efavirenz peaks in the ASD- and PM spectra were presented with lower intensities and less details. Furthermore, in the ASD spectrum, the efavirenz peak at 3313 cm^−1^ disappeared and a shift and broadening of the peak at 1744 cm^−1^ was observed in both the ASD- and PM spectra (Figure 5b). These observations might be related to interactions between efavirenz and the ModE copolymer.

In contrast to celecoxib, as well as efavirenz formulations, fenofibrate formulations demonstrated very weak drug–polymer interactions. The FT-IR spectrum of the fenofibrate-ModE ASD showed no obvious differences compared to that of the corresponding physical mixture across the entire scanning range (Figure 5c). Only the characteristic peak of fenofibrate at 2983 cm^−1^ disappeared in both spectra, which is most probably due to the lower drug concentration in the PM, as well as in ASD when compared to the pure drug (Figure 5c).

### 3.10. Saturation Solubility Studies

The saturation solubility (48 h, 20 °C) of celecoxib, efavirenz, and fenofibrate in water was determined for the pure drugs and all ASDs (Table 7). Since the three compounds exhibit pH-independent solubility under physiological conditions of the gastrointestinal tract, solubility tests in water were considered sufficient for initial screening and assessment of the suitability of different polymers for the preparation of ASDs with the desired performance [27,28]. Results of the solubility studies indicated very low aqueous solubilities for the pure drugs (Table 7). By contrast, in most cases, the preparation of ASDs succeeded in considerably improving the water solubility of the drugs. However, some ASDs did not present the desired solubility improvement. The AQOAT^®^ AS-MMP ASD, for instance, presented with low saturation solubilities of all three drugs (Table 7). The ASDs based on Kollidon^®^ 17 PF showed solubility-enhancing effects in the case of fenofibrate only. High saturation solubilities for celecoxib and efavirenz were achieved with Soluplus^®^ ASDs, while in the case of Kollidon^®^ VA 64 ASDs, the highest solubilities of efavirenz and fenofibrate were observed. The solubility enhancing effect of ModE or EPO in ASDs was not as pronounced as that of Soluplus^®^ in the case of celecoxib, and in the case of efavirenz or fenofibrate, they were also not as pronounced as that of Kollidon^®^ VA 64 (Table 7), but was well within the range of results observed for Affinisol^®^ HPMC 100 LV ASDs of the three drugs.

### 3.11. Dissolution Studies

To get a first idea about the later in vivo performance of the formulations, dissolution of the drug-loaded ASDs was studied in USP apparatus II using 500 mL of 0.1 M hydrochloric acid (HCl) as the dissolution medium. For comparison, dissolution of the unprocessed drug substances was studied in the same test setup.

**Figure 6:**
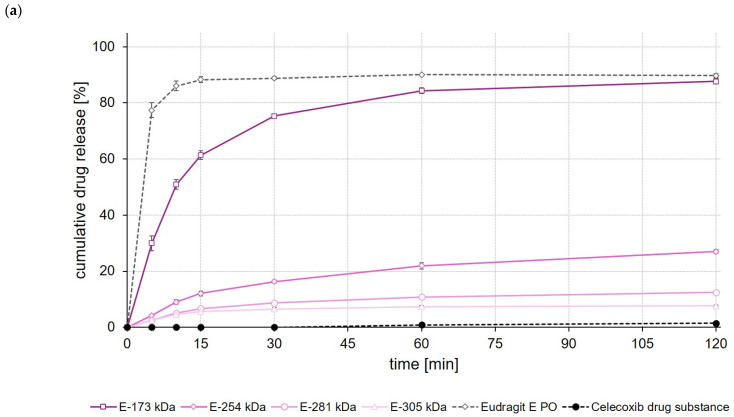

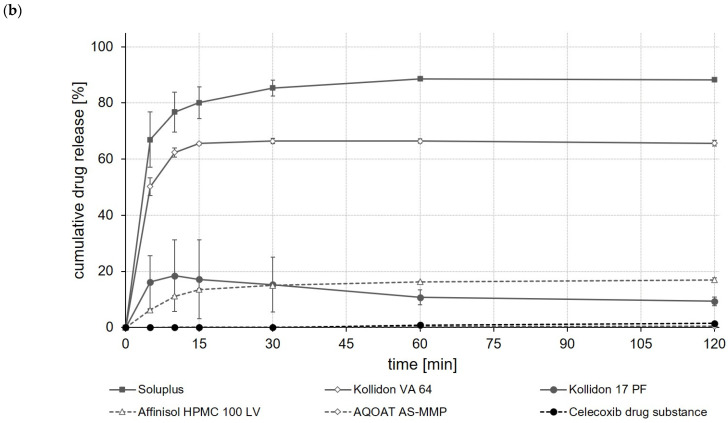
Dissolution profiles of celecoxib drug substance and celecoxib ASDs based on ModE and EPO (**a**), as well as celecoxib drug substance and celecoxib ASDs based on other marketed (co-)polymers (**b**) in 500 mL 0.1 M HCl in USP apparatus II. Each value designates the mean ± S.D. (n = 3).

Looking first at the results of the release studies with celecoxib, the ASD formulations based on EPO, Soluplus^®^ and E-173 kDa showed the highest drug release of 90% after 120 min (Figure 6a,b). The ASD formulation prepared with Kollidon^®^ VA 64 released 65% of the incorporated celecoxib dose during the same test period, while all other ASDs showed significantly (*p* < 0.05) lower celecoxib release after 120 min (Figure 6a,b).

**Figure 7:**
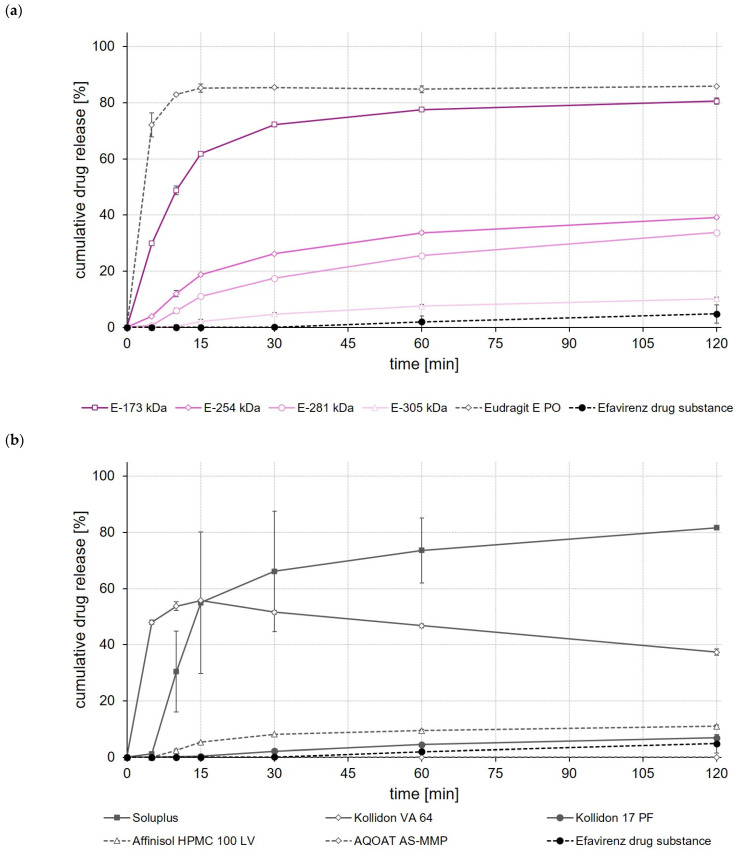
Dissolution profiles of efavirenz drug substance and efavirenz ASDs based on ModE and EPO (**a**), as well as efavirenz drug substance and efavirenz ASDs based on other marketed (co-)polymers (**b**) in 500 mL 0.1 M HCl in USP apparatus II. Each value designates the mean ± S.D. (n = 3).

As with the celecoxib ASDs, the highest drug release was achieved when EPO, Soluplus^®^ and E-173 kDa copolymers were used to prepare ASDs (Figure 7a,b). These ASDs released 80% to 85% of the incorporated efavirenz dose within 120 min. The Kollidon^®^ VA 64-based ASD formulation showed, by contrast, a typical “spring and parachute” dissolution behavior, as the drug concentration in the medium started to slowly decrease again after a rapid release of about 55% of the efavirenz dose contained in the Kollidon^®^ VA 64 ASD (Figure 7b).

**Figure 8:**
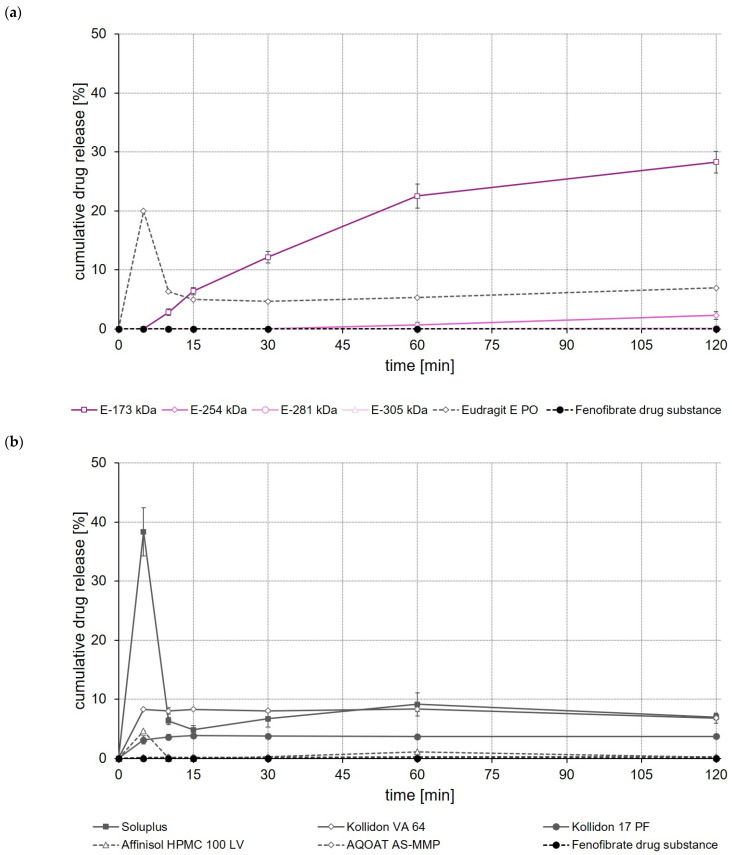
Dissolution profiles of fenofibrate drug substance and fenofibrate ASDs based on ModE and EPO (**a**), as well as fenofibrate drug substance and fenofibrate ASDs based on other marketed (co-)polymers (**b**) in 500 mL 0.1 M HCl in USP apparatus II. Each value designates the mean ± S.D. (n = 3).

In the case of fenofibrate, a significantly (*p* < 0.05) higher fenofibrate release was observed after a test duration of 120 min for the E-173 kDa ASD than for all other ASDs (Figure 8a). Whereas within the same test duration, without an apparent equilibrium already having been reached, about 30% of the incorporated fenofibrate dose was released from the E-173 kDa ASD, not more than 10% of the fenofibrate dose was released from all other ASDs (Figure 8a,b). In contrast to the ASDs prepared from Soluplus^®^, EPO and Affinisol^®^ HPMC 100 LV—which showed pronounced supersaturation effects shortly after the start of the release experiment but also precipitation quickly after reaching the supersaturated state—no supersaturation occurred, but robust fenofibrate release was observed for the E-173 kDa formulation.

When comparing the rate and extent of drug release of the ASDs prepared from different ModE types, the release of celecoxib, efavirenz, and fenofibrate was found to decrease with the higher M_w_ or higher T_g_ of the copolymer used (Figure 6a, Figure 7a and Figure 8a). An impact of the copolymers’ M_w_ on celecoxib release (dissolution rate of the drug substance was inversely proportional with M_w_ of the polymer used for ASD preparation) was also observed by Knopp et al. [29] using ASDs based on polyvinylpyrrolidone of different M_w_. With decreasing M_w_, the flexibility of a (co-)polymer usually increases while its viscosity decreases [29]. Both effects can make a positive contribution to the solubilization of a drug [29]. Results obtained in the present study suggest that the higher flexibility of the polymer chains in the E-173 kDa copolymer resulted in a good solubilization of the drugs. Overall, the E-173 kDa copolymer proved to be the most promising candidate for improving the solubility of poorly water-soluble drugs of all the (co-)polymers studied. In the case of celecoxib and efavirenz, approximately equal amounts of the drug (~80–90% of the total dose) were released from the E-173 kDa-, EPO-, and Soluplus^®^-based ASDs at the end of the experiment. However, in the case of fenofibrate, only the E-173 kDa formulation released a significant amount of the drug (~30% of the dose), while the other formulations released no more than 10% of the fenofibrate dose over the same time period; alternatively, in the case of the Soluplus^®^ formulation, although 40% of the dose was released after 5 min, this resulted in precipitation due to supersaturation and therefore only less than 10% of the fenofibrate dose remained dissolved after 10 min. In addition to the reasons already mentioned, the observed differences in drug release from ASDs prepared from different ModE copolymers could be due to the fact that the protonation of the amino groups and thus the dissolution of the copolymers takes longer with increasing M_w_, since longer polymer chains result in steric effects that could limit or impede access to the amino groups.

The results of this first set of screening experiments are quite promising. Since previous studies have shown that the drugs celecoxib, efavirenz, and fenofibrate exhibit pH-independent solubility [27,28], it could be assumed that the pH of the dissolution medium should not have a notable effect on the dissolution behavior of the drugs. For this initial set of dissolution experiments, 0.1 M HCl was selected to simulate pH conditions in the empty stomach at which all three model drugs would dissolve poorly but the new copolymers would dissolve. The addition of synthetic surfactants or bile salts, such as sodium lauryl sulfate or polyoxyethylene(20)sorbitan monooleate, as has been used in previous studies for screening drug release of enabling formulations celecoxib [29,30], fenofibrate [26,31,32] and efavirenz [23,24], was deliberately omitted in order to create worst-case conditions for the drugs under investigation and to be able to clearly map the effect of the polymers used to prepare the ASDs upon drug release. A comparison with data from the cited studies should not be made at this point because both the experimental conditions in the release studies and often also the drug loading of the formulations investigated were different.

### 3.12. Stability Studies

#### 3.12.1. Visual Appearance

After three months of storage under defined and constant conditions (30 °C/65% RH), most ASD samples did not show any significant agglomeration and could easily be shaken up again. Only Kollidon^®^ VA 64- and EPO-based ASDs were no longer easy to fluff up and, compared to all other ASDs, showed significantly larger agglomerates irrespective of the incorporated drug substance.

#### 3.12.2. Thermal Characterization of ASD via DSC Analysis after Three Months of Storage

The applied storage conditions did not have any significant impact on the T_g_ of the pure (co-)polymers (Table 6 and Table 8). Apart from Kollidon^®^ VA 64 and EPO-based ASDs incorporating fenofibrate, after three months of storage, all ASD formulations were still amorphous. Fenofibrate loaded Kollidon^®^ VA 64 and EPO-based ASDs demonstrated slight evidence of recrystallization of fenofibrate in the thermogram of the corresponding first heating cycle (data not shown here). The T_g_ of all stored ASDs was (slightly) impacted by the storage conditions (Figures S2–S7); however, except the two formulations mentioned, all others remained amorphous. The ASDs made of EPO showed low T_g_s of 40 °C, 38 °C and 34 °C, which are close to the temperature conditions applied in the storage stability studies (Table 8). These low T_g_s may allow a better movement of the polymer chains, enabling a higher motility of the drug substance and, as particularly has been seen for fenofibrate, presenting with a higher risk of recrystallization, especially when formulations are stored at elevated temperatures. After three months of storage compared to the thermograms recorded immediately after the ASDs were manufactured, significantly lower T_g_s (more than 10° C below the initial value) were observed for some of the ASDs, such as those made of celecoxib or fenofibrate and Kollidon^®^ 17 PF (Figures S5b and S7b), efavirenz and Kollidon^®^ VA 64, efavirenz or fenofibrate with AQOAT^®^ AS-MMP (Figures S6b and S7b), as well as fenofibrate with E-305 kDa (Figure S7a) (Table 6 and Table 8). By contrast, for some ASDs, such as those of efavirenz and Kollidon^®^ 17 PF (Figure S6b), and efavirenz or fenofibrate and Affinisol^®^ HPMC 100 LV (Figures S6b and S7b), T_g_s were higher after three months of storage (Table 6 and Table 8), indicating that at lower temperatures the mobility of the polymer chains was somewhat more restricted than before storage.

#### 3.12.3. Dissolution Studies after Three Months of Storage

**Figure 9:**
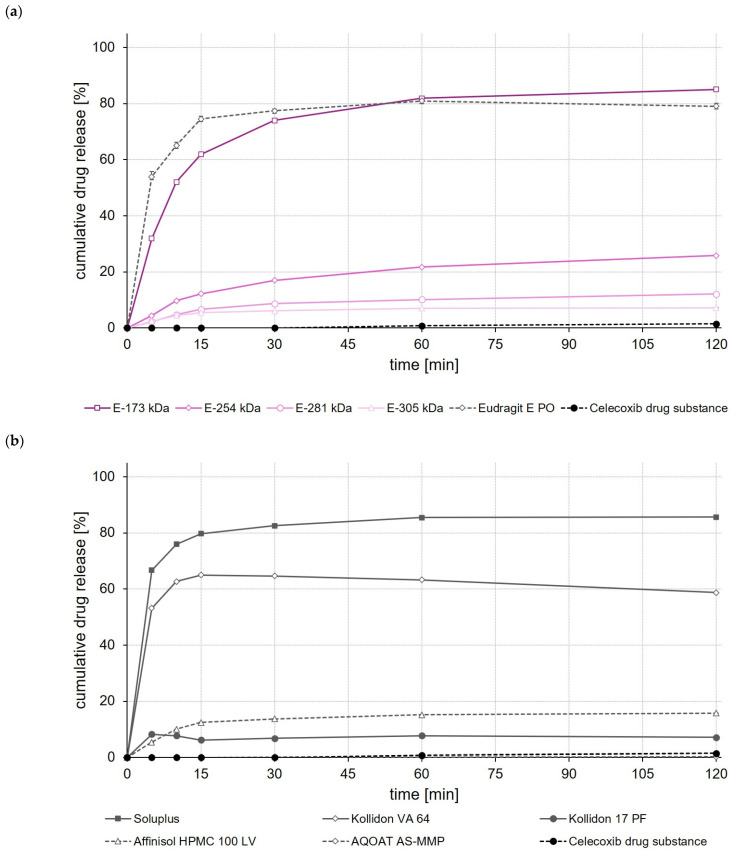
Dissolution profiles of celecoxib drug substance and celecoxib ASDs based on ModE and EPO (**a**), as well as celecoxib drug substance and celecoxib ASDs based on other marketed (co-)polymers (**b**) (after three months of storage at 30 °C/65% RH) in 500 mL 0.1 M HCl in USP apparatus II. Each value designates the mean ± S.D. (n = 3).

**Figure 10:**
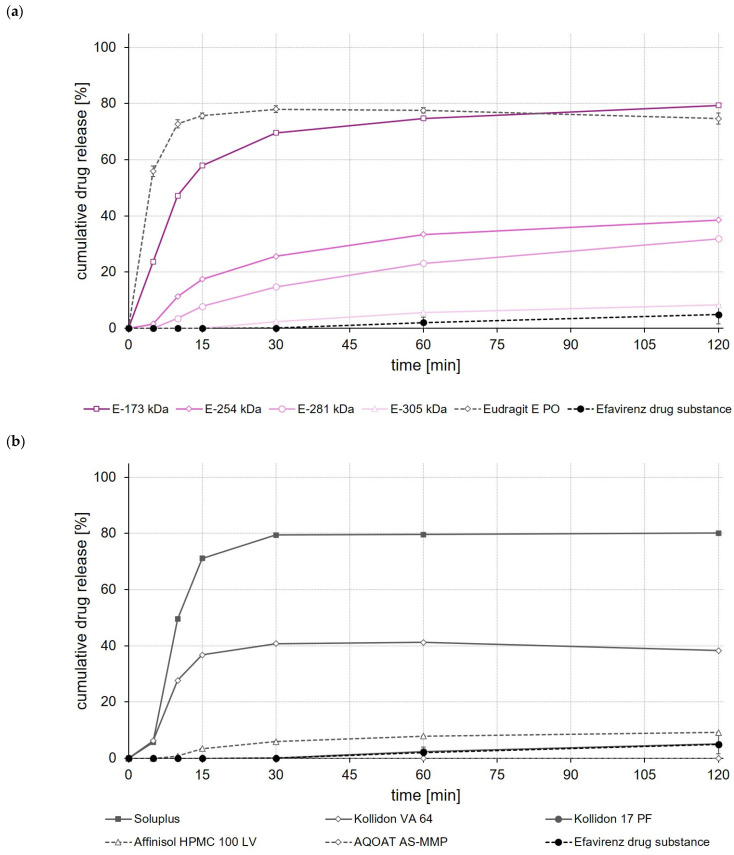
Dissolution profiles of efavirenz drug substance and efavirenz ASDs based on ModE and EPO (**a**), as well as efavirenz drug substance and efavirenz ASDs based on other marketed (co-)polymers (**b**) (after three months of storage at 30 °C/65% RH) in 500 mL 0.1 M HCl in USP apparatus II. Each value designates the mean ± S.D. (n = 3).

**Figure 11:**
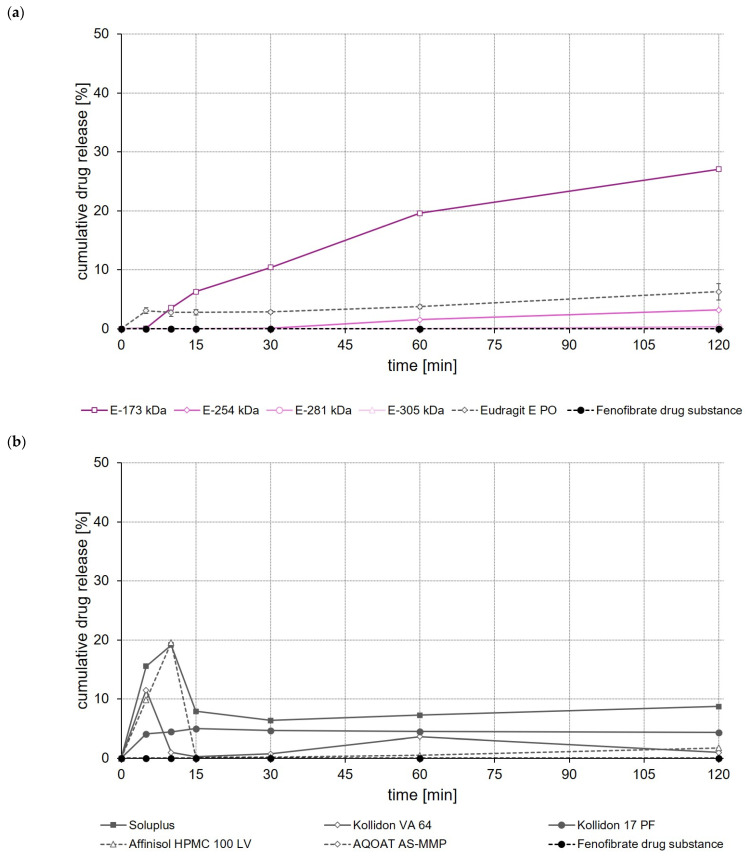
Dissolution profiles of fenofibrate drug substance and fenofibrate ASDs based on ModE and EPO (**a**), as well as fenofibrate drug substance and fenofibrate ASDs based on other marketed (co-)polymers (**b**) (after three months of storage at 30 °C/65% RH) in 500 mL 0.1 M HCl in USP apparatus II. Each value designates the mean ± S.D. (n = 3).

After three months of storage at 30 °C/65% RH, most of the ASDs showed slightly different dissolution performance (Figure 9, Figure 10 and Figure 11).

With the exception of the EPO-based ASDs (Figure 9a) and Kollidon^®^ VA 64 (Figure 9b) celecoxib, for which the amount of celecoxib released after a test duration of 120 min was significantly (*p* < 0.05) lower, after three months of storage, all other celecoxib ASDs (Figure 9a,b) showed approximately the same release behavior as immediately after manufacture. Similarly, after three months of storage, the EPO-based ASD formulation incorporating efavirenz (Figure 10a) after a test duration of 120 min revealed a significantly (*p* < 0.05) lower in vitro drug release than at the time of manufacture. In addition, the initial burst-like release of efavirenz observed within the first 10–15 min for Kollidon^®^ VA 64 ASD (Figure 10b) immediately after preparation was not as pronounced after storage. However, all other ASDs containing efavirenz (Figure 10a,b) demonstrated similar dissolution behavior as at the time of manufacture. As already suspected from the visual appearance, as well as the results of the thermal analysis after three months of storage, the Kollidon^®^ VA 64 (Figure 11b) and EPO-based (Figure 11a) ASDs showed a significantly (*p* < 0.05) lower drug release after the test period of 120 min. This may have resulted both from a reduction in the overall surface area of the formulation due to agglomeration, and from the storage-related tendency for recrystallization of fenofibrate in ASDs with the copolymers Kollidon^®^ VA 64 and EPO. An influence of storage conditions on drug release was also observed for some other fenofibrate ASDs. In particular, supersaturation effects observed for some ASD formulations in the first minutes of the release experiments were no longer as pronounced as immediately after preparation. In contrast, the dissolution profile of the ASD prepared from the E-173 kDa copolymer (Figure 11a) remained almost unchanged and still indicated a significantly (*p* < 0.05) higher amount of fenofibrate released (28% of the contained dose) than all other ASDs after an experimental period of 120 min (Figure 11b). Differences in T_g_s of all other drug–(co-)polymer combinations before and after storage were much lower, i.e., less than 5 °C in all cases.

## 4. Conclusions

By means of radical polymerization using two specific radical initiators and n-dodecylmercaptan as CTA, a novel dimethylaminopropyl methacrylamide-butyl methacrylate-methyl methacrylate copolymer (ModE) with different molecular weights was synthesized, characterized and investigated for its suitability to improve solubility and drug release of the poorly water-soluble model substances celecoxib, efavirenz and fenofibrate. For this purpose, drug-loaded ASDs were prepared with the new ModE copolymers, EPO, and several other polymers that have previously been successfully used for solubility enhancement. Based on the detailed investigation of the ASDs prepared from ModE copolymers, it can be concluded that M_w_ and T_g_ appear to have a significant impact on the drug release efficiency of poorly water-soluble drugs. The novel E-173 kDa copolymer proved to be the best candidate for improving the drug release of the chosen model drug substances. In a first set of stability experiments, ASDs prepared from this copolymer showed good storage stability, and were superior to ASDs prepared employing any other polymer used in the study with respect to the properties investigated here. Results from the present screening study therefore indicate the potential of the novel ModE copolymer for application in the field of solubility enhancement. Future studies will focus on employing several more poorly water-soluble drugs, including drugs with pH-dependent solubility, as well as increasing the drug load of the ASDs. Moreover, in vitro screening will also cover the use of biorelevant in vitro models that will allow for better estimation of the in vivo performance of the formulations.

## Figures and Tables

**Table 1:** Residual monomer content, monomer conversion rate and the final composition of ModE copolymers. Each value designates the mean (n = 3). Since S.D. was <0.04 for DMAPMA and <0.005 for all other reported values, detailed S.D. values are not shown for better overview.

Polymer	Residual Monomer Content			Monomer Conversion Rate			Final Polymer Composition		
	DMAPMA * [%]	BMA * [%]	MMA * [%]	DMAPMA [%]	BMA [%]	MMA [%]	DMAPMA [%]	BMA [%]	MMA [%]
E-173 kDa	6.48	0.002	0.022	87.04	99.99	99.91	46.55	26.74	26.71
E-254 kDa	6.79	0.002	0.037	86.42	99.99	99.85	46.38	26.83	26.79
E-281 kDa	5.59	0.002	0.034	88.82	99.99	99.86	47.06	26.49	26.45
E-305 kDa	5.78	0.002	0.045	88.44	99.99	99.82	46.96	26.54	26.50
average	6.16	0.002	0.035	87.68	99.99	99.86	46.74	26.65	26.61

* DMAPMA = dimethylaminopropyl methacrylamide, BMA = butyl methacrylate, MMA = methyl methacrylate.

**Table 2:** Residual monomer and water content after purification and drying of ModE. Each value designates the mean ± S.D. (n = 3).

Polymer	DMAPMA * [%]	BMA * [%]	MMA * [%]	Water Content after 5 Days [%] ± S.D.	Water Content after 10 Days [%] ± S.D.
E-173 kDa	0.016 ± 0.001	<0.002 ± 0	<0.002 ± 0	1.11 ± 0.05	0.60 ± 0.07
E-254 kDa	0.025 ± 0.002	<0.002 ± 0	<0.002 ± 0	1.31 ± 0.08	0.90 ± 0.03
E-281 kDa	0.028 ± 0.002	<0.002 ± 0	<0.002 ± 0	1.10 ± 0.05	0.90 ± 0.04
E-305 kDa	0.042 ± 0.003	<0.002 ± 0	<0.002 ± 0	1.11 ± 0.07	0.80 ± 0.06

* DMAPMA = dimethylaminopropyl methacrylamide, BMA = butyl methacrylate, MMA = methyl methacrylate.

**Table 3:** M_n_, M_w_ and PDI of the ModE copolymers determined via gel permeation chromatography (GPC) analysis. Each value designates the mean ± S.D. (n = 3).

Concentration of n-Dodecylmercaptan [%]	Number-Average Molecular Weight (M_n_) [kDa]	Weight-Average Molecular Weight (M_w_) [kDa]	Polydispersity Index (PDI)
1.5	41.5 ± 1.8	173 ± 0.5	4.17 ± 0.09
0.9	64.1 ± 0.4	254 ± 0.5	3.97 ± 0.02
0.5	71.7 ± 1.3	281 ± 1.0	3.92 ± 0.05
0.3	81.4 ± 0.1	305 ± 0.5	3.75 ± 0.01

**Table 4:** Flowability data of ModE considering different molecular weights (M_w_) and EPO. Each value designates the mean ± S.D. (n = 3).

	E-173 kDa	E-254 kDa	E-281 kDa	E-305 kDa	EPO *
**polymer [g]**	99.90 ± 0.45	99.17 ± 0.54	99.35 ± 0.55	99.87 ± 0.33	99.65 ± 0.41
**flow time [s]**	30.51 ± 0.60	25.37 ± 0.71	24.09 ± 0.40	23.11 ± 1.46	-
**flow rate [g/s]**	3.28 ± 0.06	3.91 ± 0.13	4.13 ± 0.09	4.34 ± 0.29	-
**slope angle [°]**	34.58 ± 0.43	31.33 ± 0.52	31.87 ± 0.54	32.20 ± 1.00	-

* ground to a particle size of approximately 0.25 mm applying EUDRAGIT^®^ E 100.

**Table 5:** Composition and hot melt extrusion process parameters of ASDs incorporating celecoxib/efavirenz/fenofibrate.

Polymer	Drug Load [%]	Extrusion Temperature [°C]	Torque [N·cm]	Screw Speed [rpm]
Soluplus^®^	5.1/6.3/4.2	150/150/150	130/105/95	200/200/200
Kollidon^®^ VA 64	5.1/6.3/4.2	160/165/170	70/90/65	200/200/200
Kollidon^®^ 17 PF	5.1/6.3/4.2	180/180/180	55/85/65	200/200/200
AQOAT^®^ AS-MMP	5.1/6.3/4.2	170/175/175	140/200/140	200/200/200
E-173 kDa	5.1/6.3/4.2	150/160/160	200/185/180	200/200/200
E-254 kDa	5.1/6.3/4.2	165/160/165	190/180/180	200/200/200
E-281 kDa	5.1/6.3/4.2	160/160/160	200/200/200	200/200/200
E-305 kDa	5.1/6.3/4.2	165/165/165	230/200/220	200/200/200
Affinisol^®^ HPMC 100 LV	5.1/6.3/4.2	165/165/170	150/150/120	100/100/100
EUDRAGIT^®^ E PO	5.1/6.3/4.2	150/150/150	50/45/45	200/200/200

**Table 6:** Glass transition temperatures (T_g_) of pure (co-)polymers and amorphous solid dispersions (ASDs) incorporating celecoxib, efavirenz or fenofibrate. Each value designates the mean ± S.D. (n = 3).

Polymer	T_g_ (Polymer) [°C]	T_g_ (Celecoxib ASD) [°C]	T_g_ (Efavirenz ASD) [°C]	T_g_ (Fenofibrate ASD) [°C]
Soluplus^®^	70 ± 0	67 ± 1	69 ± 0	62 ± 1
Kollidon^®^ VA 64	107 ± 1	100 ± 2	100 ± 1	92 ± 2
Kollidon^®^ 17 PF	136 ± 3	126 ± 3	96 ± 1	90 ± 3
AQOAT^®^ AS-MMP	113 ± 1	106 ± 1	100 ± 2	101 ± 1
E-173 kDa	77 ± 1	77 ± 1	76 ± 1	74 ± 1
E-254 kDa	85 ± 2	79 ± 0	78 ± 1	77 ± 1
E-281 kDa	89 ± 0	84 ± 1	83 ± 2	76 ± 2
E-305 kDa	91 ± 1	84 ± 1	82 ± 1	80 ± 2
Affinisol^®^ HPMC 100 LV	103 ± 2	90 ± 0	87 ± 0	84 ± 1
EUDRAGIT^®^ E PO	42 ± 1	41 ± 1	40 ± 1	34 ± 2

**Table 7:** Saturation solubility (48 h) of celecoxib, efavirenz, and fenofibrate pure drugs and ASDs in water at 20 °C. Each value designates the mean ± S.D. (n = 3).

Sample	Saturation Solubility of Celecoxib [µg/mL]	Saturation Solubility of Efavirenz [µg/mL]	Saturation Solubility of Fenofibrate [µg/mL]
Drug substance	0.6 ± 0.1	0.7 ± 0	0.1 ± 0
Soluplus^®^ ASD	34.4 ± 0.3	8.8 ± 0	1.0 ± 0
Kollidon^®^ VA 64 ASD	2.5 ± 0.1	15.5 ± 0.1	8.5 ± 0
Kollidon^®^ 17 PF ASD	0.6 ± 0	0.5 ± 0	2.3 ± 0
AQOAT^®^ AS-MMP ASD	0.7 ± 0	0.4 ± 0	0.1 ± 0
E-173 kDa ASD	3.5 ± 0.3	2.1 ± 0.2	0.9 ± 0.3
E-254 kDa ASD	3.0 ± 0.1	1.9 ± 0.1	1.2 ± 0.4
E-281 kDa ASD	2.7 ± 0.2	1.8 ± 0.2	0.8 ± 0.2
E-305 kDa ASD	2.1 ± 0.2	1.1 ± 0.1	0.6 ± 0.1
Affinisol^®^ HPMC 100 LV ASD	3.6 ± 0.2	1.7 ± 0.1	1.7 ± 0
EUDRAGIT^®^ E PO ASD	2.6 ± 0.3	1.8 ± 0.1	0.4 ± 0

**Table 8:** Glass transition temperature (T_g_) of ASDs incorporating celecoxib, efavirenz or fenofibrate after three months of storage. Each value designates the mean ± S.D. (n = 3).

Polymer	T_g_ (Polymer) [°C]	T_g_ (Celecoxib ASD) [°C]	T_g_ (Efavirenz ASD) [°C]	T_g_ (Fenofibrate ASD) [°C]
Soluplus^®^	70 ± 1	66 ± 2	65 ± 1	62 ± 3
Kollidon^®^ VA 64	106 ± 0	99 ± 3	86 ± 2	92 ± 0
Kollidon^®^ 17 PF	135 ± 2	88 ± 1	103 ± 0	78 ± 2
AQOAT^®^ AS-MMP	111 ± 1	103 ± 2	84 ± 2	86 ± 2
E-173 kDa	78 ± 1	78 ± 3	74 ± 1	70 ± 1
E-254 kDa	84 ± 1	79 ± 2	77 ± 0	77 ± 0
E-281 kDa	89 ± 1	85 ± 1	82 ± 1	78 ± 3
E-305 kDa	91 ± 0	85 ± 2	80 ± 1	70 ± 1
Affinisol^®^ HPMC 100 LV	103 ± 3	91 ± 1	98 ± 2	94 ± 2
EUDRAGIT^®^ E PO	41 ± 1	40 ± 2	38 ± 2	34 ± 1

## Data Availability

Not applicable.

## Supplementary Materials

The following supporting information can be downloaded at: https://www.mdpi.com/article/10.3390/polym14071281/s1. Figure S1. Results from isothermal TGA of ModE copolymers at 150 °C (a) and 165 °C (b); Figure S2. DSC thermograms of celecoxib, ModE copolymers, EPO and the corresponding ASDs (a), and celecoxib and other marketed (co-)polymers used in the study, and the corresponding ASDs (b); Figure S3. DSC thermograms of efavirenz, ModE copolymers, EPO and the corresponding ASDs (a), and efavirenz and other marketed (co-)polymers used in the study, and the corresponding ASDs (b); Figure S4. DSC thermograms of fenofibrate, ModE copolymers, EPO and the corresponding ASDs (a), and fenofibrate and other marketed (co-)polymers used in the study, and the corresponding ASDs (b); Figure S5. DSC thermograms (after three months of storage at 30 °C/65% RH) of celecoxib, ModE copolymers, EPO and the corresponding ASDs (a), and celecoxib and other marketed (co-)polymers used in the study, and the corresponding ASDs (b); Figure S6. DSC thermograms (after three months of storage at 30 °C/65% RH) of efavirenz, ModE copolymers, EPO and the corresponding ASDs (a), and efavirenz and other marketed (co-)polymers used in the study, and the corresponding ASDs (b); Figure S7. DSC thermograms (after three months of storage at 30 °C/65% RH) of fenofibrate, ModE copolymers, EPO and the corresponding ASDs (a), and fenofibrate and other marketed (co-)polymers used in the study, and the corresponding ASDs (b).
