# Supplementary material for: A Novel Aminomethacrylate-Based Copolymer for Solubility Enhancement—From Radical Polymer Synthesis to Manufacture and Characterization of Amorphous Solid Dispersions
Source: Polymers (Basel). 2022 Mar 22;14(7):1281. doi: 10.3390/polym14071281 (PMC9003068; doi:10.3390/polym14071281)
Supplement: Supplementary file 1 [file polymers-14-01281-s001.zip › polymers-1631686-supplementary.pdf]

polymers

MDPI

Supplementary materials

A Novel Aminomethacrylate-Based Copolymer for Solubility Enhancement—From Radical Polymer Synthesis to Manufacture and Characterization of Amorphous Solid Dispersions

Fabian-Pascal Schmied [1,2], Alexander Bernhardt [2], Christian Moers [2], Christian Meier [3], Thomas Endres [2] and Sandra Klein [1,*]

[1] University of Greifswald, Department of Pharmacy, Institute of Biopharmaceutics and Pharmaceutical Technology, Felix-Hausdorff-Straße 3, 17489 Greifswald, Germany; fabian-pascal.schmied@stud.uni-greifswald.de (F.-P.S.); sandra.klein@uni-greifswald.de (S.K.)
[2] Evonik Operations GmbH, Research, Development & Innovation, Kirschenallee, 64293 Darmstadt, Germany; fabian-pascal.schmied@evonik.com (F.-P.S.); alexander.bernhardt@evonik.com (A.B.); christian.moers@evonik.com (C.M.); thomas.endres@evonik.com (T.E.)
[3] Evonik Operations GmbH, Research, Development & Innovation, Rodenbacher Chaussee 4, 63457 Hanau, Germany; christian.meier@evonik.com (C.M.)
* Correspondence: sandra.klein@uni-greifswald.de; Tel.: +49-3834-420-4897

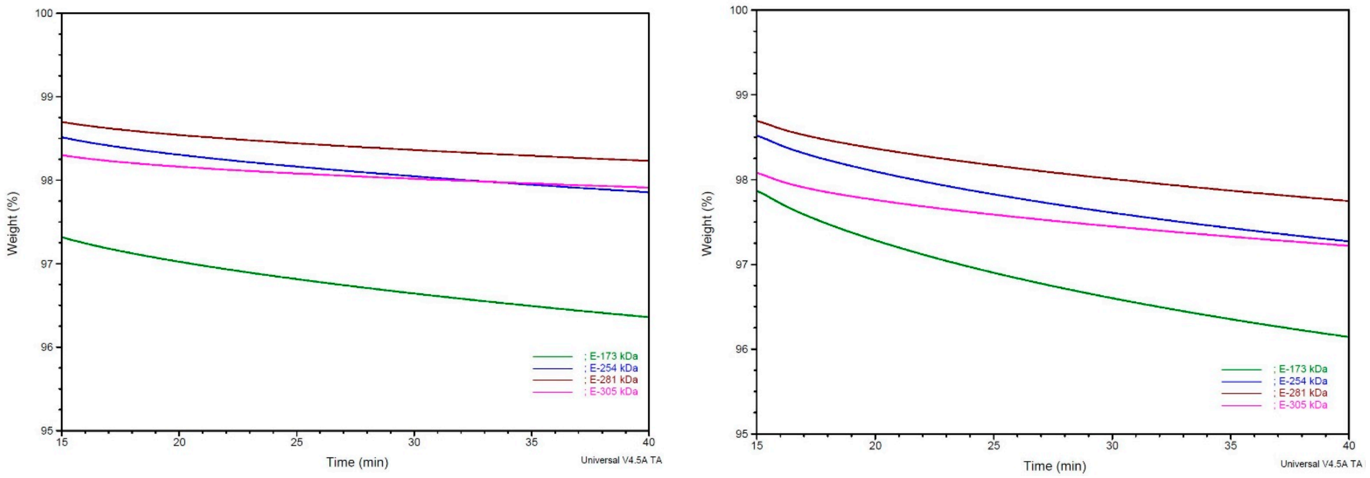

(**a**) (**b**)

Figure S1. Results from isothermal TGA of ModE copolymers at 150 °C (**a**) and 165 °C (b).

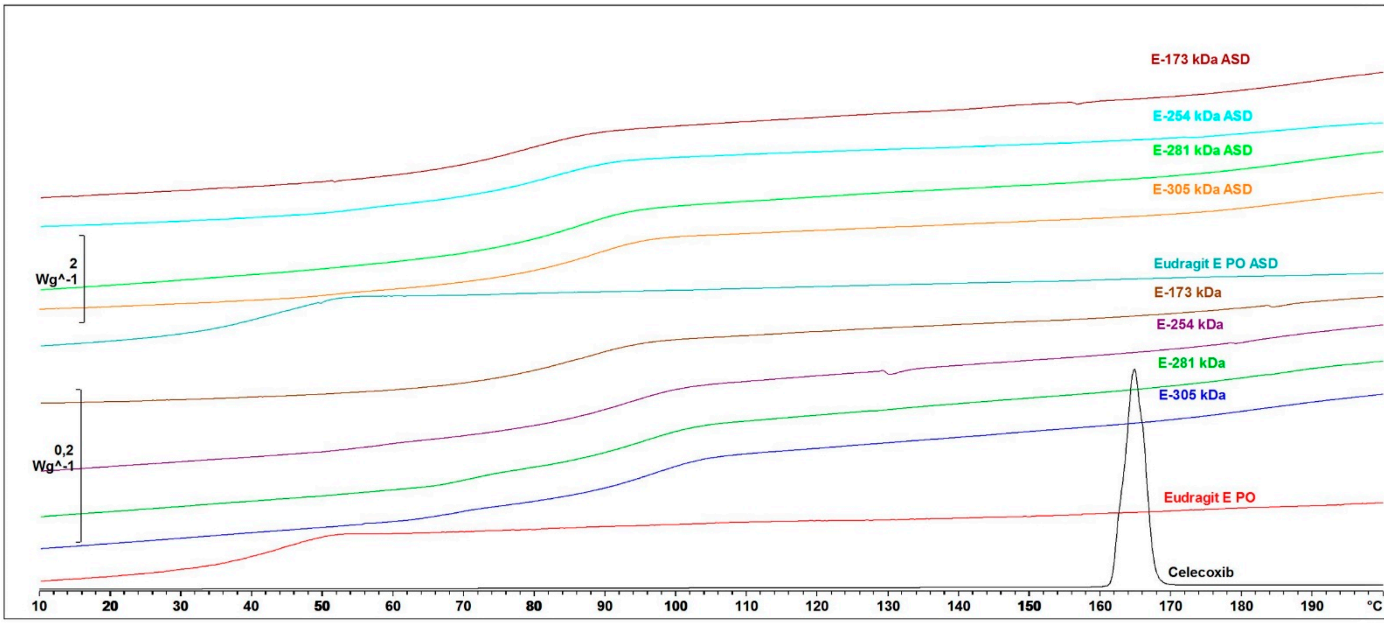

(**a**)

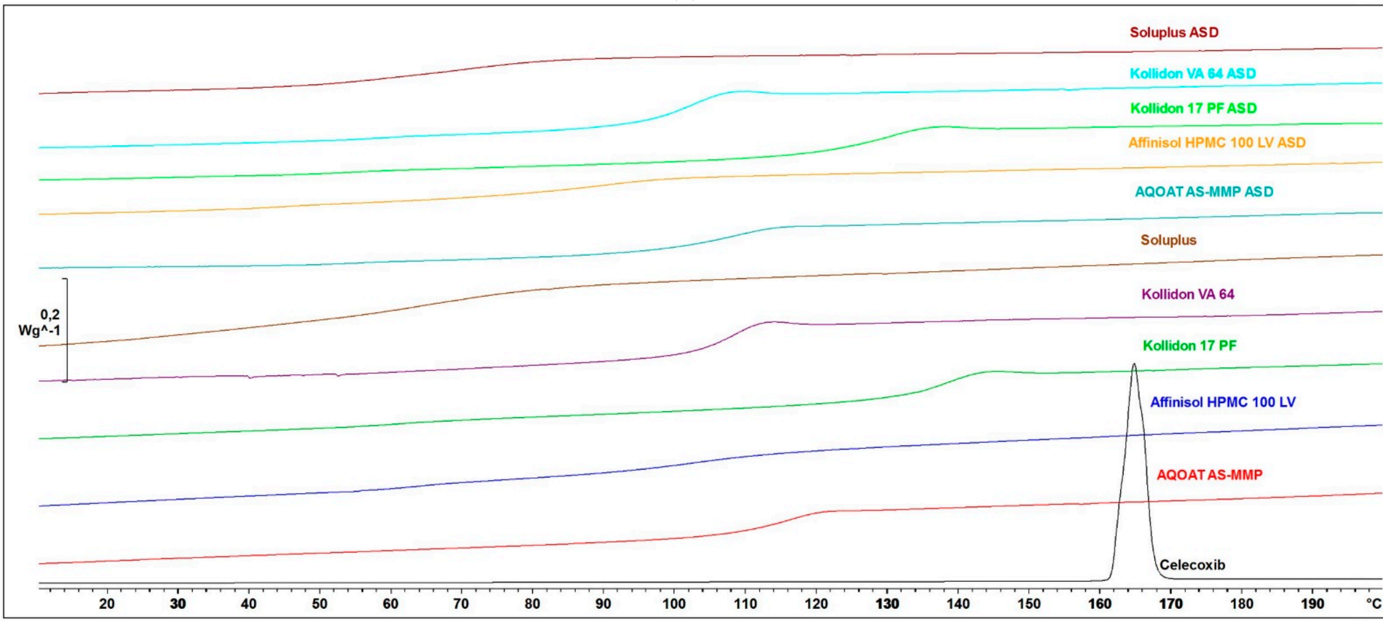

(**b**)

Figure S2. DSC thermograms of celecoxib, ModE copolymers, EPO and the corresponding ASDs (**a**), and celecoxib and other marketed (co-)polymers used in the study, and the corresponding ASDs (**b**).

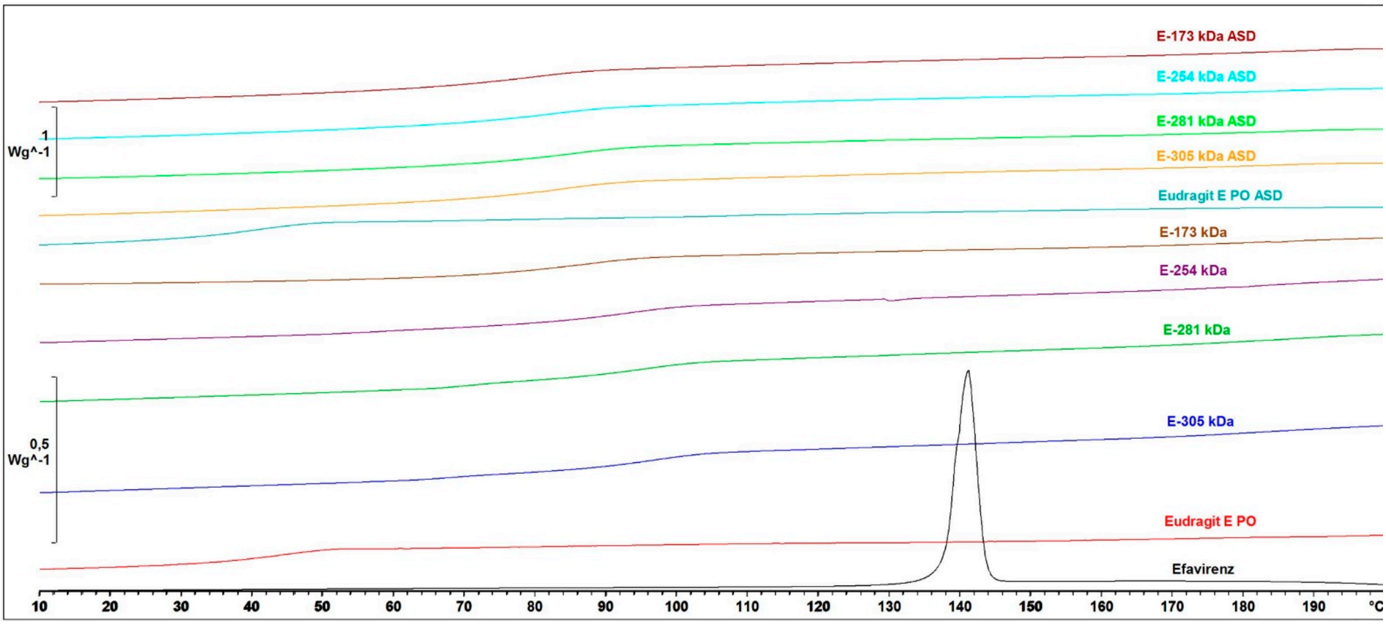

(**a**)

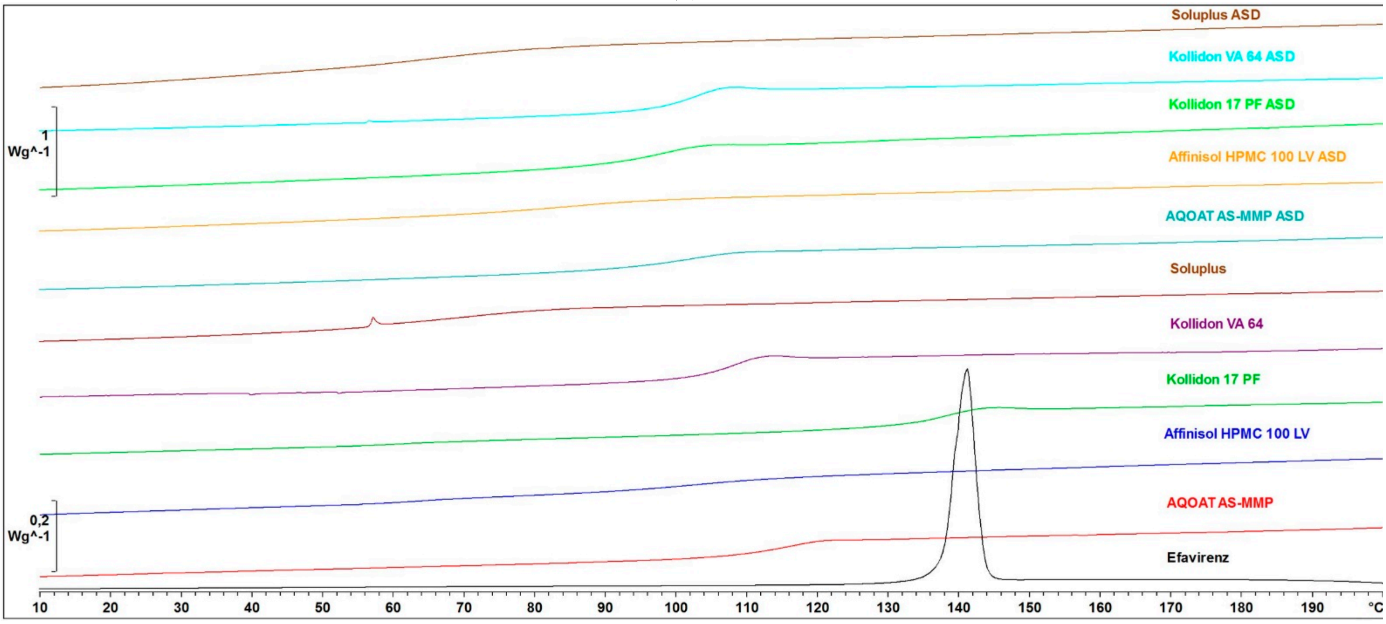

(**b**)

Figure S3. DSC thermograms of efavirenz, ModE copolymers, EPO and the corresponding ASDs (**a**), and efavirenz and other marketed (co-)polymers used in the study, and the corresponding ASDs (**b**).

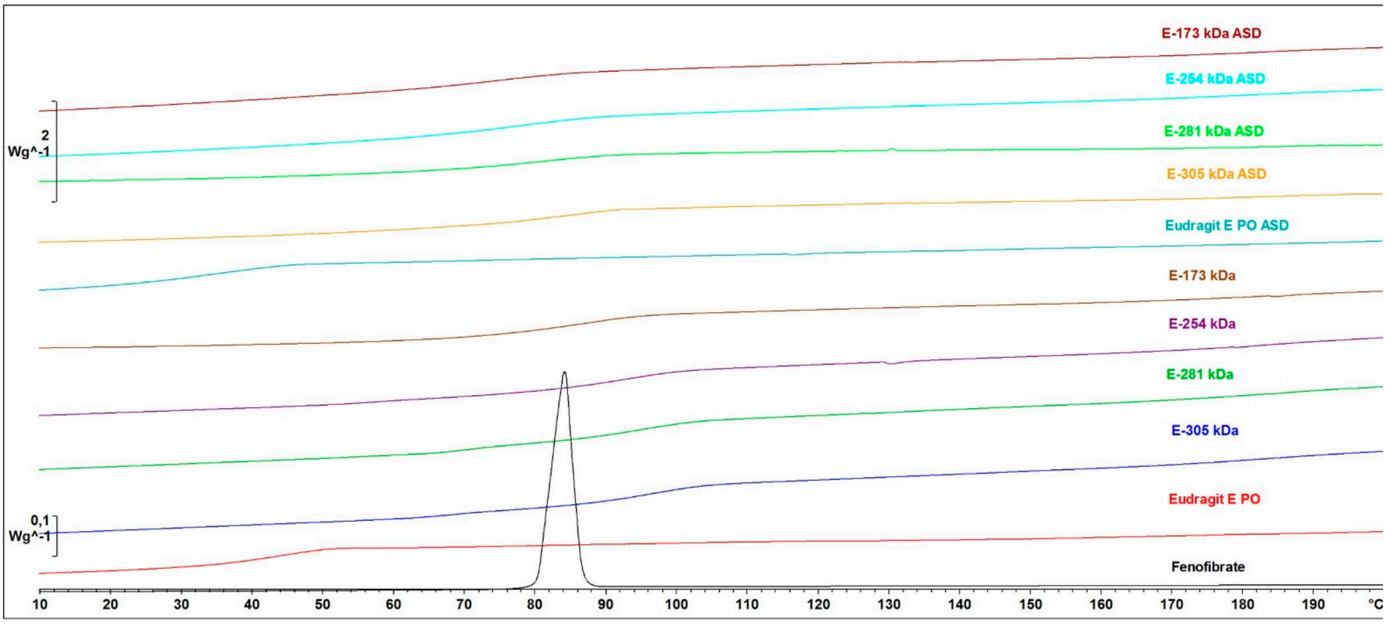

(**a**)

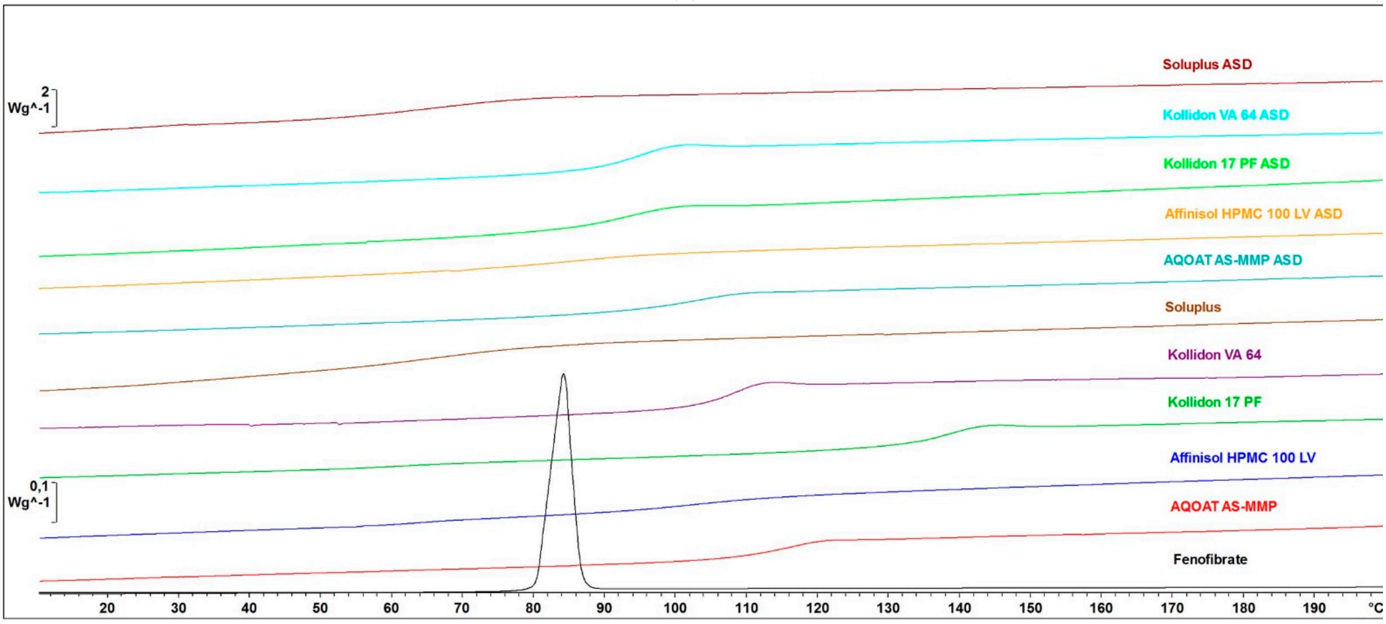

(**b**)

Figure S4. DSC thermograms of fenofibrate, ModE copolymers, EPO and the corresponding ASDs (**a**), and fenofibrate and other marketed (co-)polymers used in the study, and the corresponding ASDs (**b**).

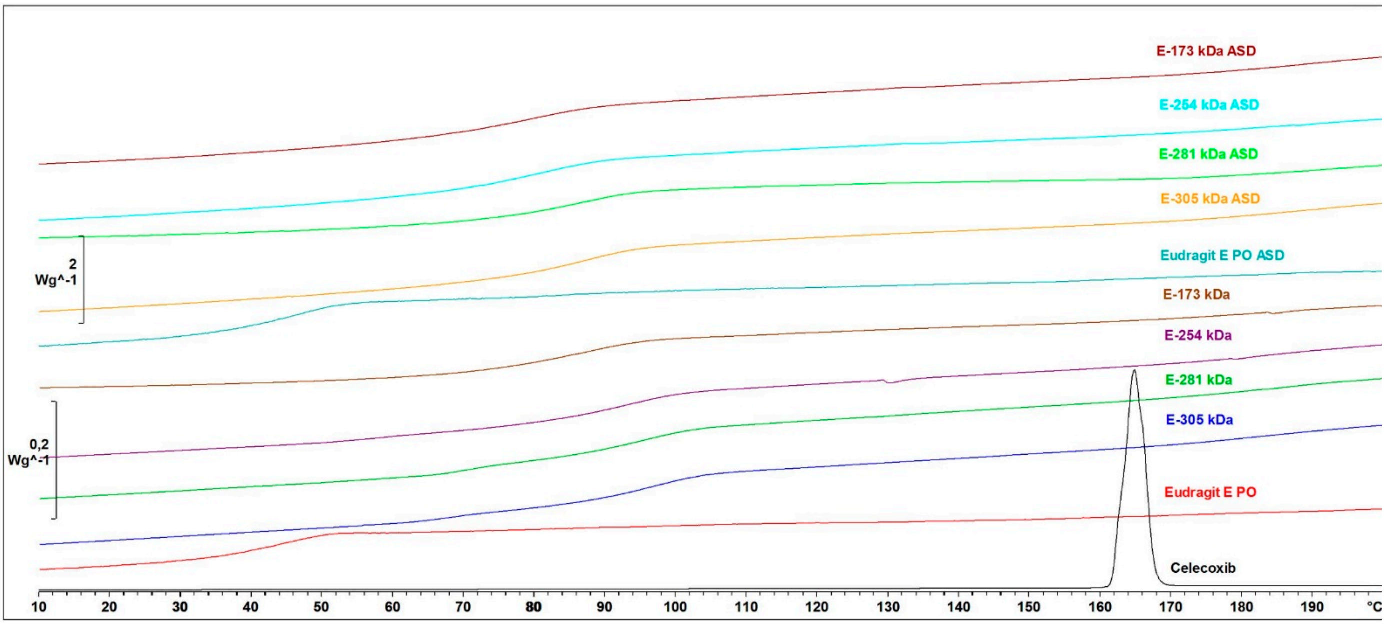

(**a**)

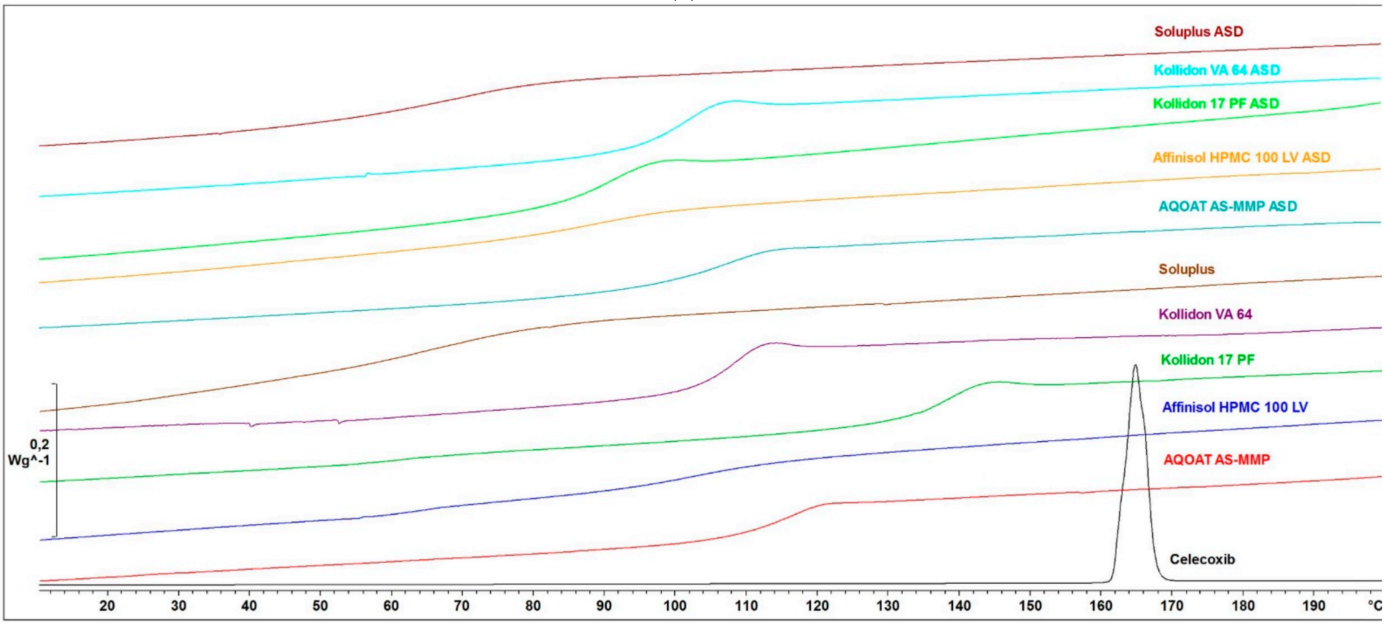

(**b**)

Figure S5. DSC thermograms (after three months of storage at 30 °C/65% RH) of celecoxib, ModE copolymers, EPO and the corresponding ASDs (**a**), and celecoxib and other marketed (co-)polymers used in the study, and the corresponding ASDs (**b**);.

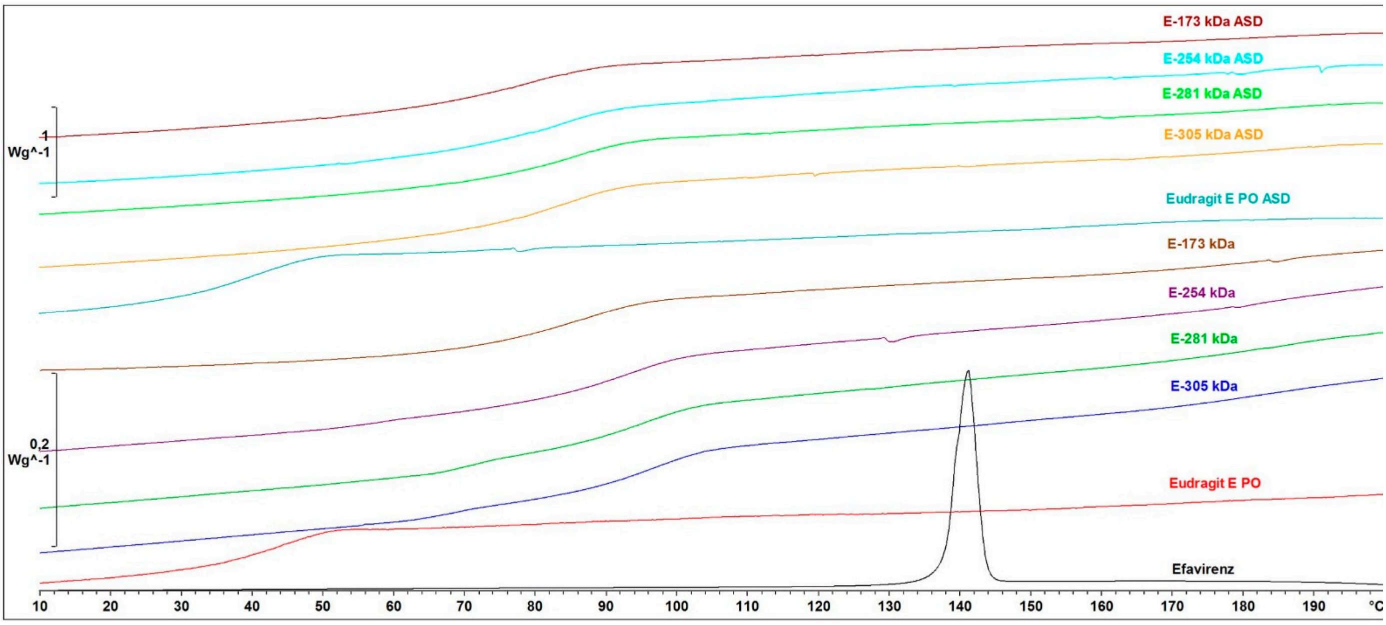

(**a**)

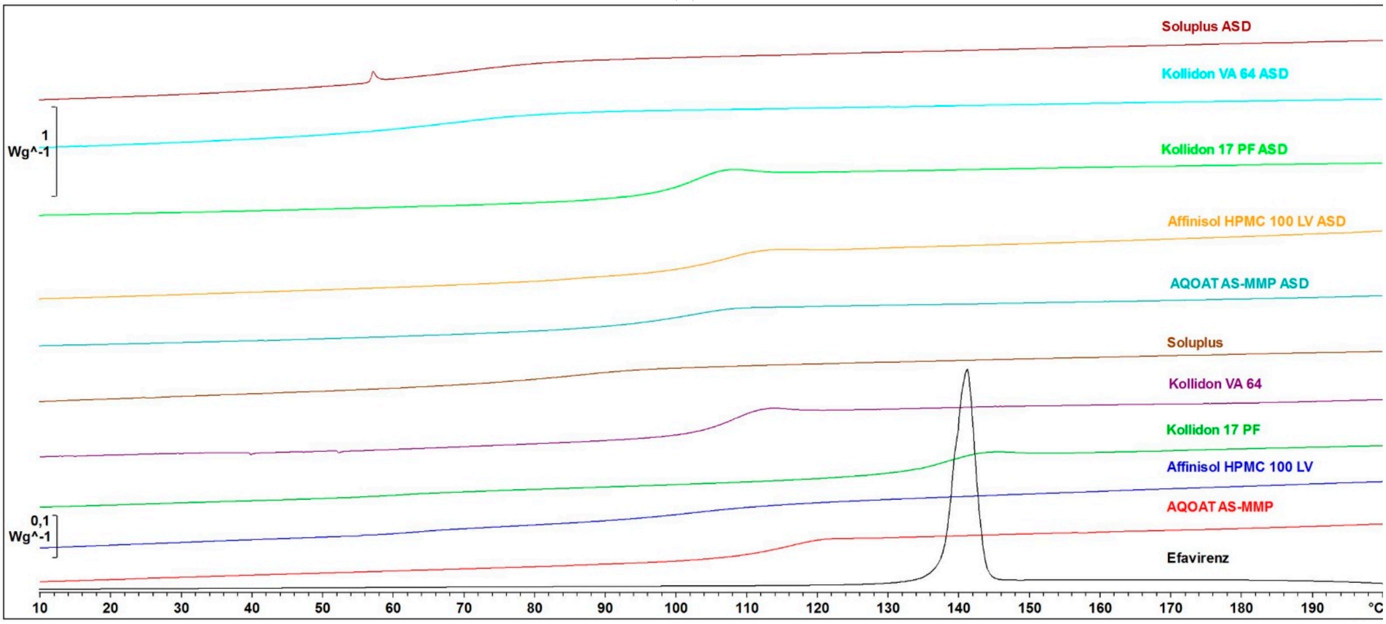

(**b**)

Figure S6. DSC thermograms (after three months of storage at 30 °C/65% RH) of efavirenz, ModE copolymers, EPO and the corresponding ASDs (**a**), and efavirenz and other marketed (co-)polymers used in the study, and the corresponding ASDs (**b**).

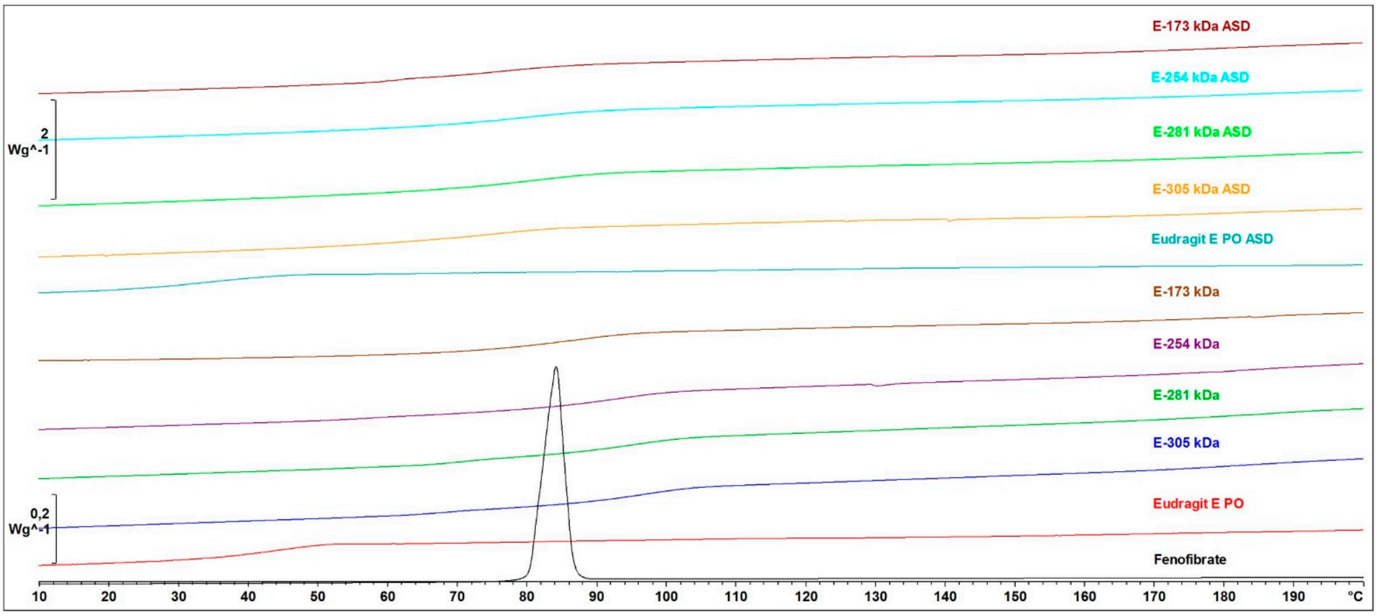

(**a**)

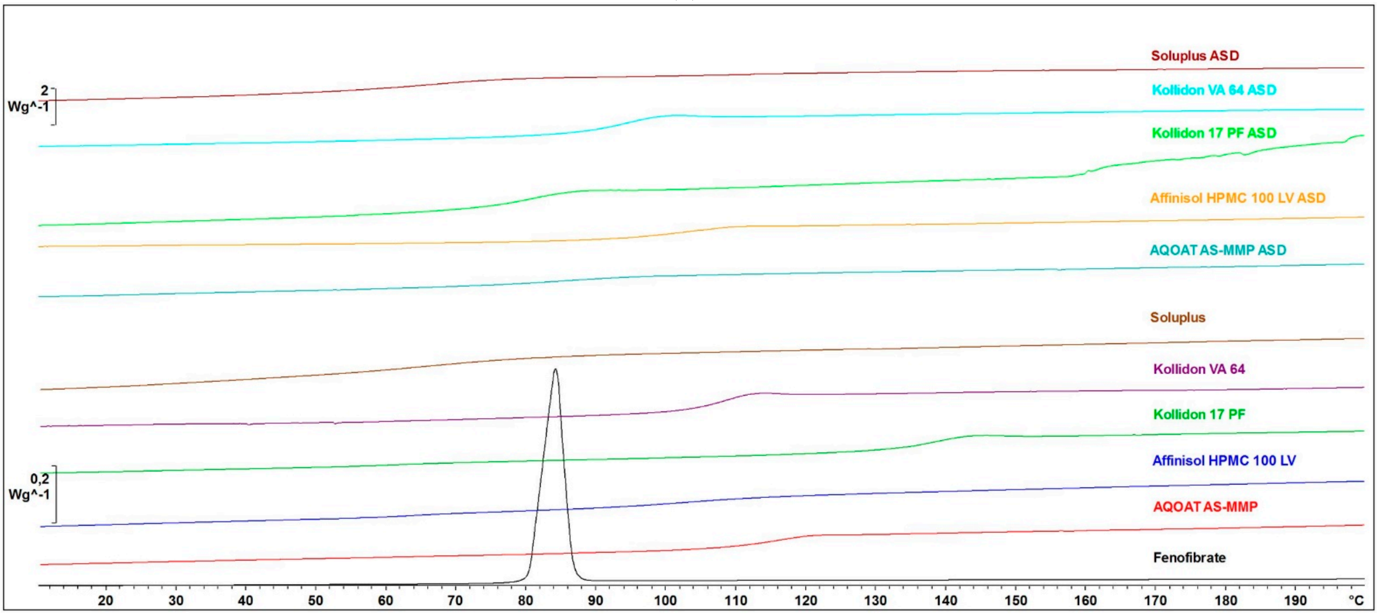

(**b**)

Figure S7. DSC thermograms (after three months of storage at 30 °C/65% RH) of fenofibrate, ModE copolymers, EPO and the corresponding ASDs (**a**), and fenofibrate and other marketed (co-)polymers used in the study, and the corresponding ASDs (**b**).
